# Development and Validation of an Interpretable Machine Learning‐Based Clinical Prediction Model for Short‐Term Mortality in Intracerebral Hemorrhage With Thrombocytopenia: A Multicenter Study

**DOI:** 10.1002/cns.71067

**Published:** 2026-07-30

**Authors:** Dachang Qiu, Guangwei Li, Ze Wang, Lin Wang, Lanlan Wang, Yongfei Dong

**Affiliations:** ^1^ The Affiliated Hospital of Qingdao University Qingdao Medical College, Qingdao University Qingdao Shandong PR China; ^2^ Institute of Artificial Intelligence, Hefei Comprehensive National Science Center Hefei Anhui PR China; ^3^ Wuxi Ninth People's Hospital Affiliated to Soochow University Medical College, Soochow University Suzhou Jiangsu PR China; ^4^ Faculty of Medical and Health Sciences University of Auckland Auckland New Zealand; ^5^ Department of Geriatrics, The First Affiliated Hospital of USTC, Division of Life Sciences and Medicine University of Science and Technology of China Hefei Anhui PR China; ^6^ Department of Neurosurgery, The First Affiliated Hospital of USTC, Division of Life Sciences and Medicine University of Science and Technology of China Hefei Anhui PR China

**Keywords:** clinical prediction model, intracerebral hemorrhage, machine learning, thrombocytopenia

## Abstract

**Background:**

Intracerebral hemorrhage (ICH) with thrombocytopenia is associated with poor outcomes, but early risk prediction tools for this subgroup are limited. We aimed to develop and externally validate an interpretable machine learning model for predicting 28‐day all‐cause mortality after ICU admission.

**Methods:**

Internal data were derived from MIMIC‐III/IV, eICU, and NWICU, and external validation data from a single tertiary academic teaching hospital in China. The internal cohort was randomly split 7:3 into internal training set and internal test set. Feature selection, hyperparameter optimization, and training of five machine learning models were performed in the internal training set. Performance was evaluated in the internal test set and external validation cohort. SHAP analysis was used for interpretation, and a web‐based tool was developed.

**Results:**

Among 1190 included patients, 859 were in the internal cohort and 331 in the external validation cohort. Fifteen predictors were retained. LightGBM showed the best performance, with AUROCs of 0.840 and 0.764 in the internal test and external validation cohorts, respectively. Important predictors included GCS, diastolic blood pressure, glucose, and platelet count.

**Conclusion:**

The developed LightGBM model showed good internal discrimination, acceptable external discrimination, and interpretable feature contributions, supporting its potential as a complementary early risk stratification aid for patients with ICH and thrombocytopenia. Prospective multicenter validation is required before clinical implementation.

## Graphical abstract

In this study, we developed and validated a clinical prediction model for the early prediction of short‐term mortality in patients with intracerebral hemorrhage complicated by thrombocytopenia. The model demonstrated good internal discrimination and acceptable external performance and may serve as a complementary tool for individualized risk stratification pending further prospective multicenter validation.

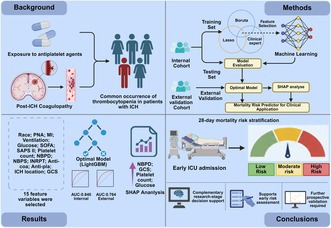

## 1 Introduction

Intracerebral hemorrhage (ICH) is a severe cerebrovascular disease with high mortality and disability [1, 2, 3]. Despite advances in critical care, effective treatments remain limited [4, 5, 6].

Platelets play a central role in hemostasis, and thrombocytopenia, defined as a platelet count < 150 × 10^9^/L in adults, may adversely affect outcomes after ICH [7, 8, 9]. Thrombocytopenia is frequently encountered in patients with ICH. In a previous multicenter ICH cohort, 294 of 2183 patients (13.5%) had thrombocytopenia, defined as a platelet count < 150 × 10^9^/L, on admission [10]. The mechanisms underlying thrombocytopenia in this population remain incompletely understood, given the complex pathophysiology of secondary brain injury after ICH [5]. This condition may result from a single factor or the combined effects of multiple clinical processes, while pre‐ICH antiplatelet exposure and post‐ICH coagulation disturbances may contribute to impaired hemostasis in clinical practice [10, 11, 12, 13, 14, 15].

Previous studies have suggested that ICH patients with thrombocytopenia may have poorer outcomes than those without this condition. A possible mechanism is that thrombocytopenia may impair local hemostasis and increase the risk of ongoing bleeding or hematoma expansion [16, 17]. Platelets not only participate in primary hemostasis but also provide a procoagulant surface for thrombin generation and fibrin formation; therefore, reduced platelet availability may compromise clot stability and facilitate continued bleeding or rebleeding [17]. In addition, platelet insufficiency may impair endothelial and blood–brain barrier repair, thereby aggravating blood–brain barrier disruption, inflammatory‐cell infiltration, cerebral edema, and secondary brain injury [10, 18, 19]. Accordingly, adverse clinical outcomes in this population warrant close clinical attention. Early mortality prediction in this subpopulation may help clinicians recognize patients who require closer monitoring and further clinical evaluation. However, existing prognostic models for ICH have rarely focused specifically on patients with concomitant thrombocytopenia.

Therefore, the present study aimed to develop and externally validate an interpretable machine learning model for early risk stratification of 28‐day all‐cause mortality after ICU admission in ICU patients with ICH and thrombocytopenia.

## 2 Methods

### 2.1 Data Sources

Data were obtained from four international public ICU databases and one tertiary academic teaching hospital in China. The public datasets included MIMIC‐III CareVue (2001–2008), MIMIC‐IV version 3.1 (2008–2022), the eICU Collaborative Research Database (2014–2015), and the Northwestern ICU database (2020–2022). Overlapping MIMIC‐III and MIMIC‐IV cases were excluded by the database providers [20, 21, 22, 23, 24]. The external validation cohort comprised patients treated from 2015 to 2025 in the Department of Neurocritical Care and Traumatic Brain Injury, The First Affiliated Hospital of the University of Science and Technology of China (USTC). This study was reported following the Transparent Reporting of a multivariable prediction model for Individual Prognosis Or Diagnosis‐Artificial Intelligence (TRIPOD+AI) guidelines [25].

### 2.2 Inclusion and Exclusion Criteria

**FIGURE 1:**
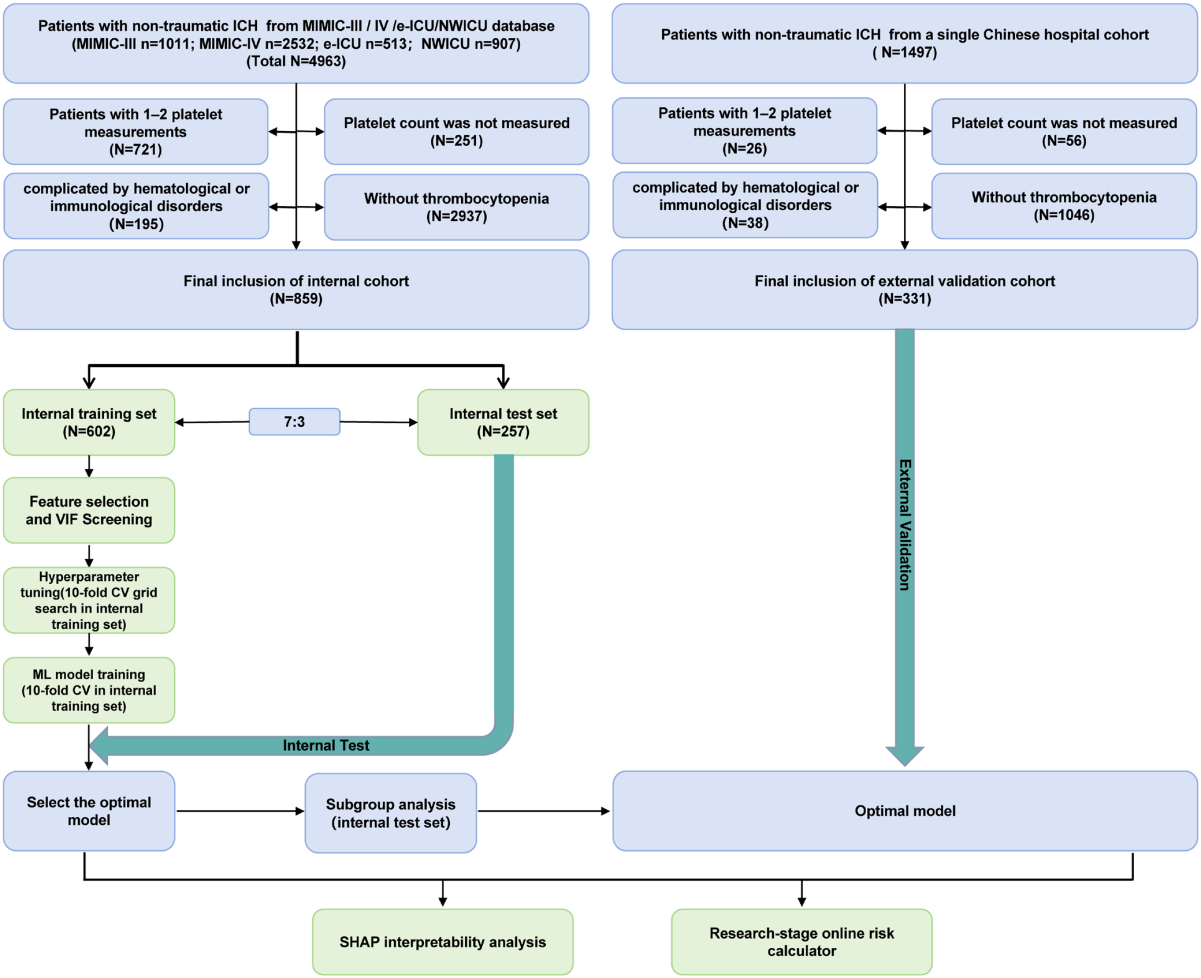
Study flow chart. AUC, area under the curve; CV, cross‐validation; DCA, decision curve analysis; ICH, intracerebral hemorrhage; PR‐Curve, precision‐recall curve; ROC, receiver operating characteristic curve.

Patients with non‐traumatic ICH were identified using ICD‐9 (431) and ICD‐10 (I61.0–I61.9) diagnostic codes. Inclusion criteria: (1) Age > 18 years; (2) Diagnosis of non‐traumatic ICH upon ICU admission; (3) The first platelet count measured after ICU admission was < 150 × 10^9^/L, and at least one of the subsequent two measurements was also < 150 × 10^9^/L; (4) ICU stay > 24 h. Exclusion criteria: (1) No platelet count recorded during ICU stay; (2) Fewer than three platelet count measurements during ICU stay; (3) Concurrent hematological or autoimmune disease, including immune thrombocytopenic purpura (ITP), acute leukemia (AL), systemic lupus erythematosus (SLE), or myelodysplastic syndromes (MDS). Patients from MIMIC‐III/IV, eICU‐CRD, and NWICU constituted the internal cohort, whereas patients from the single Chinese hospital constituted the external validation cohort. The patient selection flowchart is presented in Figure 1.

### 2.3 Definition of ICH Patients Complicated With Thrombocytopenia and Outcome Variables

Thrombocytopenia was defined as a first platelet count < 150 × 10^9^/L after ICU admission and at least one of the subsequent two measurements also < 150 × 10^9^/L. All platelet measurements used to define persistent thrombocytopenia were obtained within the first 24 h after ICU admission. The primary outcome was all‐cause death within 28 days after ICU admission. Patients alive beyond 28 days after ICU admission were classified as survivors. Across all cohorts, patients discharged alive before day 28 were also classified as survivors only when no death was documented within the 28‐day follow‐up window in the available mortality records.

### 2.4 Data Extraction

Extracted variables included demographics, vital signs, GCS, SOFA, SAPS II, laboratory tests, baseline treatments, comorbidities, ICH location, hematoma volume, and 28‐day outcomes. All candidate predictors were based on measurements obtained within 24 h of ICU admission. Hematoma location and volume were obtained from primary diagnoses and radiology reports. However, because many MIMIC radiology reports used qualitative descriptors rather than exact volumes, hematoma volume was highly incomplete. Duplicate public‐database records were removed using Subject ID and Stay ID.

### 2.5 Missing Value Imputation

Variables with more than 30% missing values were excluded from model development. Remaining missing values were imputed using random forest multiple imputation. The internal cohort was split into internal training set and internal test set before imputation. The imputation model was fitted using only the internal training set and without outcome information, then applied unchanged to the internal test set and external validation cohort. Missingness and imputation parameters are reported in Tables S1 and S2.

### 2.6 Feature Selection

All variables included in the prediction model were obtained within 24 h after ICU admission. Feature selection was performed exclusively within the internal training set, and neither the internal test set nor the external validation cohort was used for feature selection. LASSO regression with 10‐fold cross‐validation and the Boruta algorithm were used to identify candidate predictors, followed by clinical review. Three physicians with associate professor rank or above independently reviewed baseline variables based on clinical relevance, existing evidence, availability at the prediction time point, and relevance to ICH severity, thrombocytopenia, coagulation status, systemic illness, respiratory support, and neurocritical care prognosis. A variable was added only when all three experts agreed, and the experts were blinded to model performance. Expert‐added variables and their clinical rationale are provided in Table S3. Variance inflation factors were assessed to remove multicollinear variables, using a threshold of 5.

### 2.7 Model Development and Validation

#### 2.7.1 Hyperparameter Tuning

Five machine learning algorithms were evaluated: random forest (RF), logistic regression (LR), light gradient boosting machine (LightGBM), support vector machine (SVM), and multilayer perceptron (MLP). Hyperparameters were optimized by grid search with 10‐fold cross‐validation in the internal training set.

#### 2.7.2 Model Development and Internal Validation

Each model was trained in the internal training set using its optimal hyperparameters and 10‐fold cross‐validation. Discrimination was assessed using AUROC with 95% confidence intervals (CIs), calibration using the Brier score, calibration intercept, and calibration slope, and classification performance using the Youden‐index threshold. AUROCs were compared using DeLong's test. The Youden‐index threshold was determined in the internal training set and then fixed for evaluation in the internal test and external validation cohorts. Platt scaling was applied only to the final LightGBM model; it was fitted in the internal training set using model‐derived logits and observed outcomes, then applied unchanged to the internal test set and external validation cohort. Bootstrap resampling was used to estimate CIs for calibration metrics where feasible (Table S4). Calibration curves, precision‐recall (PR) curves, decision curve analysis (DCA), and confusion matrices were also generated. The final model was compared with GCS, SOFA, and SAPS II, and subgroup analyses assessed its performance in the internal test set.

#### 2.7.3 External Validation of the Optimal Model

The optimal model was externally validated in the external validation cohort using 15 features. AUROC, calibration curves, DCA, PR curves, and confusion matrices were computed. Model discrimination was further benchmarked against GCS, SOFA, and SAPS II in the external validation cohort using DeLong's test.

### 2.8 SHAP (Shapley Additive Explanations) Analysis

SHAP analysis was used to examine global feature importance and individual‐level contributions to model predictions.

### 2.9 Clinical Application of the Model

A web‐based risk prediction tool was developed using the R Shiny platform to facilitate early risk identification for all‐cause mortality within 28 days after ICU admission among patients with ICH and thrombocytopenia.

### 2.10 Sensitivity Analyses

Four sensitivity analyses were performed. First, a LightGBM model incorporating the 15 primary predictors plus admission hematoma volume was retrained in the hematoma‐volume‐complete internal cohort and evaluated in the hematoma‐volume‐complete external validation cohort. Second, the original 15‐predictor LightGBM model was applied without refitting to the hematoma‐volume‐complete external validation subgroup. Third, we included all patients with platelet testing within 24 h of ICU admission who met the thrombocytopenia criterion (< 150 × 10^9^/L), regardless of measurement frequency, to assess model stability and potential selection bias related to platelet‐testing frequency. In addition, we performed a sensitivity analysis using a model developed from the algorithm‐selected variable set to compare its performance with that of the main model incorporating expert‐added variables. For retrained models, predictor definitions, preprocessing, hyperparameter optimization, 10‐fold cross‐validation, Platt calibration, and external validation procedures matched the primary analysis.

### 2.11 Statistical Analysis

A study reproducibility table is provided in Table S5. Statistical analyses were performed using R Software (Version 4.4.1). Nested cross‐validation was not performed. Hyperparameter optimization was conducted exclusively within the internal training set using 10‐fold cross‐validation. Final model performance was then independently evaluated in the internal test set and the external validation cohort, neither of which was used for feature selection, hyperparameter tuning, model training, threshold selection, or probability calibration. Continuous variables were assessed for normality and summarized as medians with interquartile ranges when skewed; categorical variables were summarized as counts and percentages. Between‐group comparisons used the Mann–Whitney *U* test or chi‐square test, as appropriate. DeLong's test was used to compare AUROCs, with multiple‐comparison correction to reduce false‐positive findings. Two‐sided *p* < 0.05 was considered statistically significant.

## 3 Results

### 3.1 Baseline Characteristics of Participants

A total of 1190 patients with ICH and thrombocytopenia were included: 859 in the internal cohort and 331 in the external validation cohort. The 28‐day mortality rates were 27.36% and 25.68%, respectively. Univariate comparisons showed that non‐survivors had higher SOFA (both *p* < 0.05), SAPS II (both *p* < 0.001), and glucose levels (both *p* < 0.01) in both cohorts. In contrast, age (internal cohort: *p* = 0.941; external validation cohort: *p* = 0.312), GCS (internal cohort: *p* = 0.176; external validation cohort: *p* = 0.147), and NBPS (internal cohort: *p* = 0.143; external validation cohort: *p* = 0.518) were not significantly different between survivors and non‐survivors in either cohort. NBPD differed significantly in the internal cohort (*p* = 0.037) but not in the external validation cohort (*p* = 0.528). Detailed baseline comparisons are presented in Table 1 and Table S6. No correction for multiple comparisons was applied to baseline characteristics, as the baseline tables were merely for descriptive purposes and univariate analysis results were not utilized for model development or validation. The internal cohort was randomly divided into an internal training set and an internal test set at a 7:3 ratio, with no significant differences between subsets (Table S7).

**TABLE 1:** Key baseline characteristics of patients.

Variable	Internal cohort (internal training set + internal test set) *N* = 859 (602 + 257)			External validation cohort *N* = 331		
	Non‐death within 28 days *N* = 624	Death within 28 days *N* = 235	*p*	Non‐death within 28 days *N* = 246	Death within 28 days *N* = 85	*p*
Age	70.00 (62.00, 80.00)	72.00 (59.00, 81.00)	0.941	69.00 (63.00, 80.00)	74.00 (60.00, 83.00)	0.312
SOFA	5.00 (3.00, 6.00)	6.00 (4.00, 9.00)	< 0.001	4.00 (3.00, 7.00)	5.00 (4.00, 10.00)	0.002
SAPS II	35.00 (28.00, 43.00)	42.00 (33.00, 55.00)	< 0.001	35.00 (27.00, 42.00)	41.50 (34.00, 57.00)	< 0.001
GCS	14.00 (10.00, 15.00)	14.00 (7.00, 15.00)	0.176	14.00 (11.00, 15.00)	13.00 (6.00, 15.00)	0.147
NBPS, mmHg	132.00 (120.00, 146.00)	130.00 (116.00, 145.00)	0.143	133.00 (113.00, 146.00)	133.00 (117.00, 147.00)	0.518
NBPD, mmHg	73.00 (63.00, 84.00)	69.00 (59.00, 82.00)	0.037	72.00 (63.00, 84.00)	69.00 (58.00, 89.00)	0.528
INRPT	1.20 (1.10, 1.40)	1.30 (1.10, 1.50)	0.012	1.20 (1.10, 1.40)	1.30 (1.10, 1.50)	0.013
Platelet count, K/μL	121.00 (91.00, 139.00)	123.00 (87.00, 139.00)	0.559	124.00 (96.00, 139.50)	127.75 (95.00, 142.50)	0.705
Glucose, mg/dL	126.00 (102.00, 158.50)	134.00 (116.00, 183.00)	< 0.001	124.00 (99.00, 157.00)	132.50 (117.00, 183.00)	0.003
ICH location			0.011			0.031
Basal ganglia	81.00 (12.98%)	23.00 (9.79%)		31.00 (12.60%)	5.00 (5.88%)	
Cerebral lobe	99.00 (15.87%)	30.00 (12.77%)		44.00 (17.89%)	13.00 (15.29%)	
Brainstem	6.00 (0.96%)	11.00 (4.68%)		3.00 (1.22%)	6.00 (7.06%)	
Cerebellum	44.00 (7.05%)	13.00 (5.53%)		19.00 (7.72%)	4.00 (4.71%)	
Ventricle	56.00 (8.97%)	26.00 (11.06%)		28.00 (11.38%)	11.00 (12.94%)	
Multifocal hemorrhage	6.00 (0.96%)	1.00 (0.43%)		2.00 (0.81%)	0.00 (0.00%)	
Others	332.00 (53.21%)	131.00 (55.74%)		119.00 (48.37%)	46.00 (54.12%)	
Sex			0.083			0.003
Female	239.00 (38.30%)	75.00 (31.91%)		105.00 (42.68%)	23.00 (27.06%)	
Male	385.00 (61.70%)	160.00 (68.09%)		141.00 (57.32%)	62.00 (72.94%)	
Race			0.042			0.002
White	407.00 (65.22%)	144.00 (61.28%)		9.00 (3.66%)	1.00 (1.18%)	
Black	55.00 (8.81%)	11.00 (4.68%)		14.00 (5.69%)	3.00 (3.53%)	
Asian	26.00 (4.17%)	12.00 (5.11%)		204.00 (82.93%)	77.00 (90.58%)	
Others	136.00 (21.79%)	68.00 (28.94%)		19.00 (7.72%)	4.00 (4.71%)	
HTN			0.696			0.106
No	304.00 (48.72%)	118.00 (50.21%)		115.00 (46.75%)	34.00 (40.00%)	
Yes	320.00 (51.28%)	117.00 (49.79%)		131.00 (53.25%)	51.00 (60.00%)	
PNA			0.221			0.110
No	486.00 (77.88%)	192.00 (81.70%)		194.00 (78.86%)	58.00 (68.24%)	
Yes	138.00 (22.12%)	43.00 (18.30%)		52.00 (21.14%)	27.00 (31.76%)	
T2DM			0.993			0.845
No	507.00 (81.25%)	191.00 (81.28%)		190.00 (77.24%)	65.00 (76.47%)	
Yes	117.00 (18.75%)	44.00 (18.72%)		56.00 (22.76%)	20.00 (23.53%)	
MI			0.029			0.251
No	610.00 (97.76%)	223.00 (94.89%)		239.00 (97.15%)	80.00 (94.12%)	
Yes	14.00 (2.24%)	12.00 (5.11%)		7.00 (2.85%)	5.00 (5.88%)	
COPD			0.297			0.364
No	586.00 (93.91%)	225.00 (95.74%)		232.00 (94.31%)	82.00 (96.47%)	
Yes	38.00 (6.09%)	10.00 (4.26%)		14.00 (5.69%)	3.00 (3.53%)	
Anti‐coa			0.166			0.542
No	572.00 (91.67%)	222.00 (94.47%)		228.00 (92.68%)	80.00 (94.12%)	
Yes	52.00 (8.33%)	13.00 (5.53%)		18.00 (7.32%)	5.00 (5.88%)	
Anti‐pla			0.010			0.170
No	579.00 (92.79%)	229.00 (97.45%)		224.00 (91.06%)	78.00 (91.76%)	
Yes	45.00 (7.21%)	6.00 (2.55%)		22.00 (8.94%)	7.00 (8.24%)	
CRRT			0.002			0.117
No	608.00 (97.44%)	218.00 (92.77%)		239.00 (97.15%)	79.00 (92.94%)	
Yes	16.00 (2.56%)	17.00 (7.23%)		7.00 (2.85%)	6.00 (7.06%)	
Ventilation			< 0.001			0.018
No	293.00 (46.96%)	62.00 (26.38%)		106.00 (43.09%)	20.00 (23.53%)	
Yes	331.00 (53.04%)	173.00 (73.62%)		140.00 (56.91%)	65.00 (76.47%)	

*Note:* Baseline comparisons were based on the completed imputed analysis datasets; Detailed baseline characteristics of the patients are provided in Table S6.

Abbreviations: Anti‐coa, Anticoagulation agent; Anti‐pla, Antiplatelet agent; CRRT, Continuous Renal Replacement Therapy; GCS, Glasgow Coma Scale; HTN, Hypertension; INRPT, International Normalized Ratio of Prothrombin Time; MI, Myocardial Infarction; NBPD, Non‐invasive Blood Pressure Diastolic; NBPS, Non‐invasive Blood Pressure Systolic; PNA, Pneumonia; SAPS II, Simplified Acute Physiology Score II; SOFA, Sequential Organ Failure Assessment; T2DM, Type 2 Diabetes Mellitus.

### 3.2 Feature Selection

**FIGURE 2:**
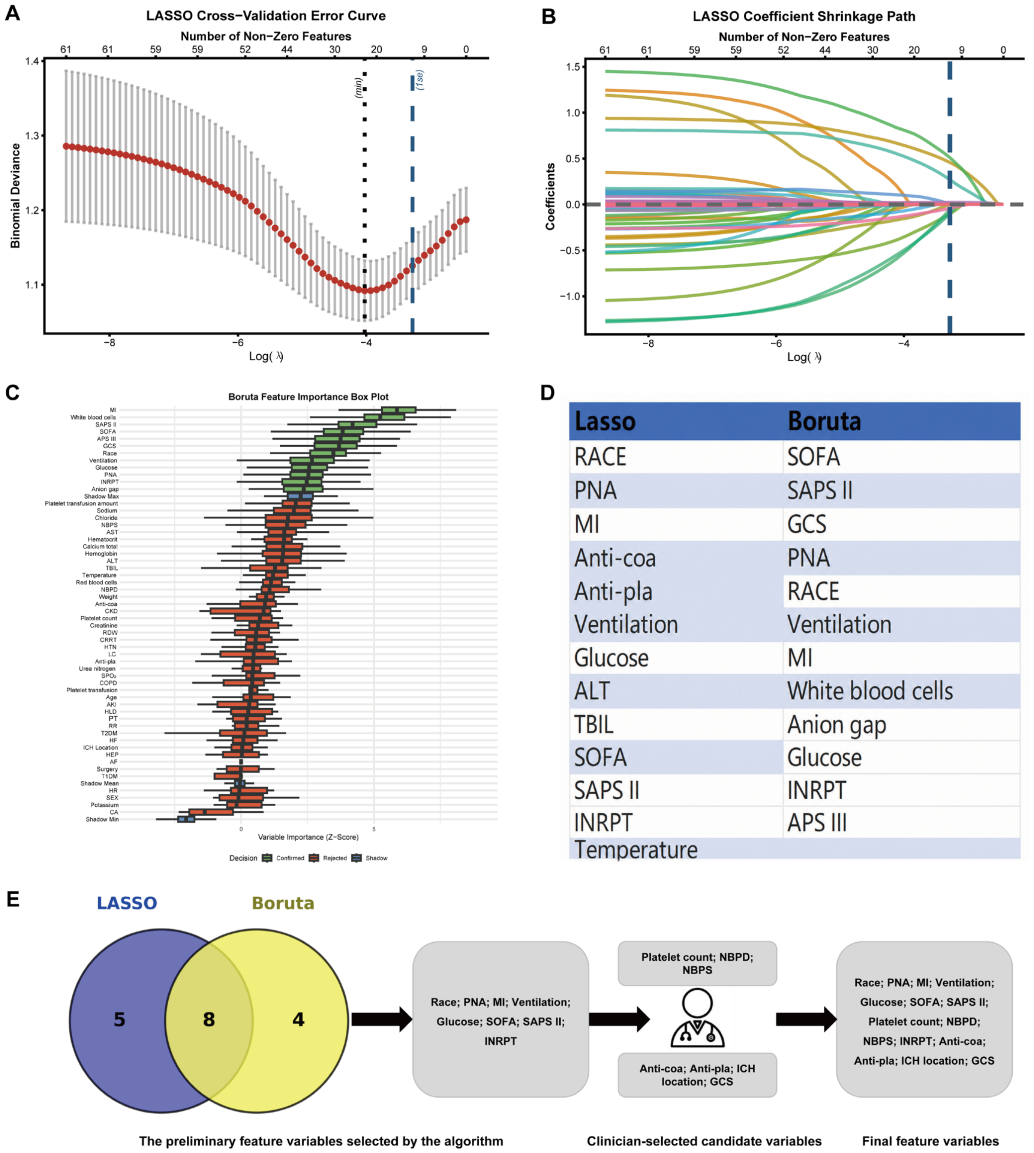
Feature selection. (A) LASSO deviance plot; (B) LASSO coefficient Shrinkage path plot; (C) Boruta feature importance boxplot; (D) Variables selected by LASSO and Boruta; (E) Flowchart of final feature inclusion; LASSO regression retained 13 variables using the *λ*_1se_ criterion. AF, atrial fibrillation; AG, anion gap; AKI, acute kidney injury; ALT, alanine aminotransferase; Anti‐coa, anticoagulant agent; Anti‐pla, antiplatelet agent; APS III, Acute Physiology Score III; AST, aspartate aminotransferase; CA, carcinoma; CKD, chronic kidney disease; COPD, chronic obstructive pulmonary disease; CRRT, continuous renal replacement therapy; GCS, Glasgow Coma Scale; HEP, hepatitis; HF, heart failure; HLD, hyperlipidemia; HR, heart rate; HTN, hypertension; ICH, intracerebral hemorrhage; INRPT, international normalized ratio of prothrombin time; LASSO, least absolute shrinkage and selection operator; LC, liver cirrhosis; MI, myocardial infarction; NBPD, non‐invasive diastolic blood pressure; NBPS, non‐invasive systolic blood pressure; PNA, pneumonia; PT, prothrombin time; RBC, red blood cell; RDW, red cell distribution width; RR, respiratory rate; SAPS II, Simplified Acute Physiology Score II; SOFA, Sequential Organ Failure Assessment; SpO_2_, peripheral oxygen saturation; T1DM, type 1 diabetes mellitus; T2DM, type 2 diabetes mellitus; TBIL, total bilirubin; WBC, white blood cell.

LASSO regression using the lambda.1se criterion retained 13 variables, and Boruta independently identified 12 variables (Tables S8 and S9, Figure 2A–C). Their overlap yielded eight core variables, and seven additional clinically relevant variables were retained after expert review, resulting in 15 final predictors (Figure 2D,E). VIF analysis showed low multicollinearity (Table S10).

### 3.3 Model Performance Evaluation

**FIGURE 3:**
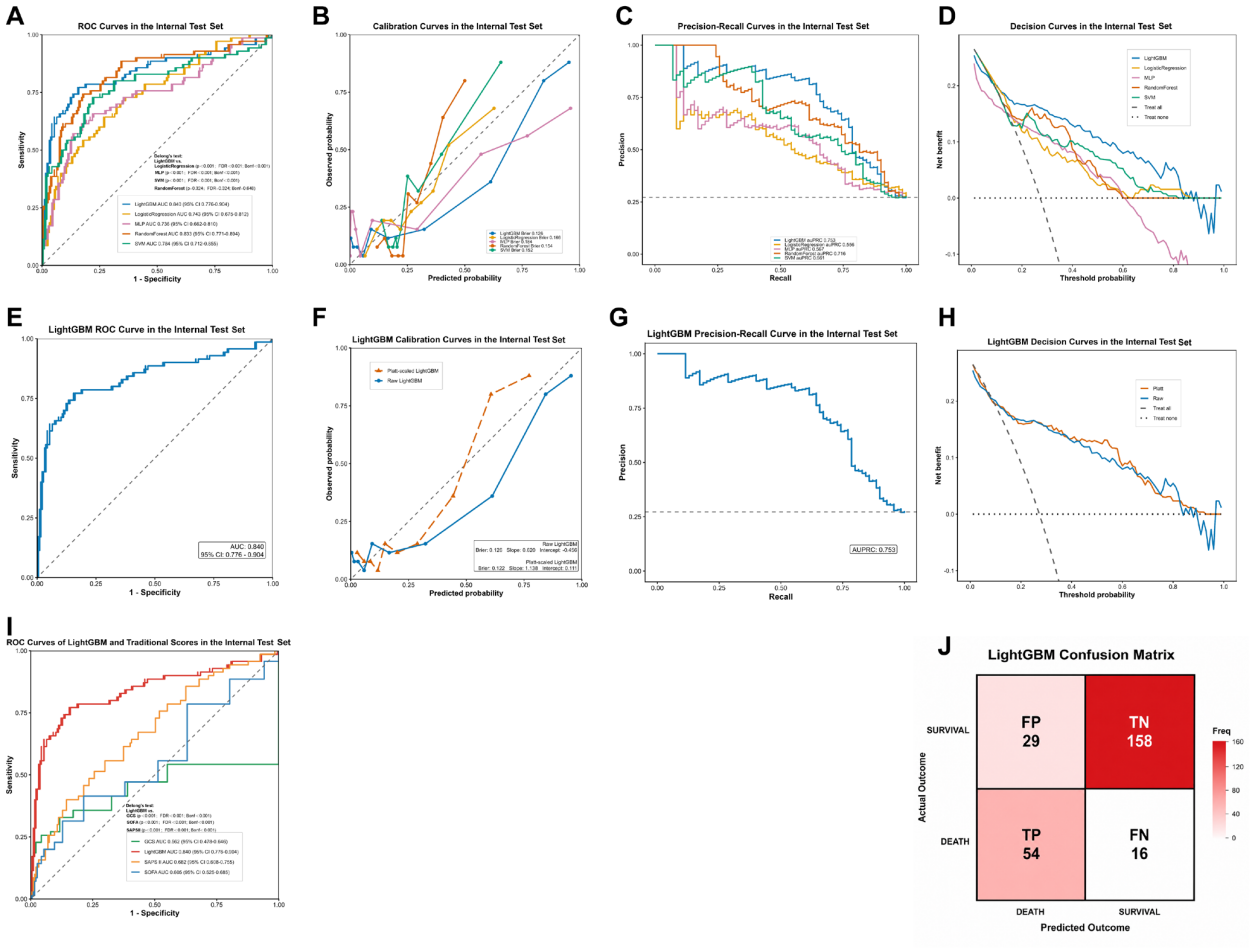
Model performance in the internal test set. (A) ROC curves of five representative models in the internal test set; (B) Calibration curves of five representative models in the internal test set; (C) PR curves of five representative models in the internal test set; (D) DCA of five representative models in the internal test set; (E) ROC curve of LightGBM in the internal test set; (F) Calibration curves of LightGBM before and after Platt scaling in the internal test set; (G) PR curve of LightGBM in the internal test set; (H) DCA of LightGBM before and after Platt scaling in the internal test set; (I) DeLong test for the predictive performance of LightGBM versus traditional clinical scores in the internal test set; (J) Confusion matrix results of LightGBM models in the internal test set. AUC, area under the curve; AUPRC, area under the precision–recall curve; AUROC, area under the receiver operating characteristic curve; CI, confidence interval; FN, false negative; FP, false positive; GCS, Glasgow Coma Scale; LightGBM, light gradient boosting machine; MLP, multilayer perceptron; ROC, receiver operating characteristic; SAPS II, Simplified Acute Physiology Score II; SOFA, Sequential Organ Failure Assessment; SVM, support vector machine; TN, true negative; TP, true positive.

Hyperparameter‐tuning results are presented in Table S11. Ten‐fold cross‐validation curves of the five representative models in the internal training set are shown in Figure S1. Among the five models, LightGBM showed the best overall performance in the internal test set, with an AUROC of 0.840 (95% CI, 0.776–0.904; Table 2, Figure 3A). LightGBM significantly outperformed LR, SVM, and MLP, but not RF (AUROC, 0.833; 95% CI, 0.771–0.894; *p* = 0.324; Table 2, Figure 3A). The model also achieved the lowest Brier score before Platt calibration (0.126), the highest AUPRC (0.753), and favorable net benefit across most threshold probabilities (Figure 3B–D). After Platt scaling, the Brier score decreased from 0.126 to 0.122, suggesting improved overall probability accuracy; however, the calibration slope increased from 0.620 to 1.138, indicating mild overcorrection in the internal test set. Because Platt scaling is a monotonic probability transformation, AUROC and threshold‐based classification metrics remained unchanged. Individual LightGBM ROC, calibration, PR, and DCA curves are shown in Figure 3E–H.

**TABLE 2:** Performance metrics of machine learning models in the internal test set.

Model	Threshold	Youden index	Accuracy	Prevalence	Recall	Precision	Specificity	F1 score	MCC	AUROC (95% CI)	Brier score	Calibration intercept	Calibration slope
LightGBM (before platt)	0.386	0.616	0.825	0.272	0.771	0.651	0.845	0.706	0.587	0.840 (0.776–0.904)	0.126	−0.456	0.62
LightGBM (after platt)	0.320	0.616	0.825	0.272	0.771	0.651	0.845	0.706	0.587	0.840 (0.776–0.904)	0.122	0.111	1.138
SVM	0.248	0.509	0.767	0.272	0.729	0.554	0.781	0.63	0.473	0.784 (0.712–0.855)	0.152	0.779	1.79
Logistic regression	0.301	0.375	0.708	0.272	0.643	0.474	0.733	0.545	0.346	0.743 (0.675–0.812)	0.166	0.136	1.14
MLP	0.420	0.443	0.77	0.272	0.614	0.573	0.829	0.593	0.434	0.736 (0.662–0.810)	0.184	−0.504	0.285
RF	0.291	0.566	0.802	0.272	0.743	0.612	0.824	0.671	0.536	0.833 (0.771–0.894)	0.154	1.452	2.638

Abbreviations: LightGBM, light gradient boosting machine; MLP, multilayer perceptron; RF, random forest; SVM, support vector machine.

Compared with traditional clinical scores, LightGBM achieved a significantly higher AUROC than GCS, SOFA, and SAPS II in the internal test set (all adjusted *p* < 0.001; Figure 3I). At the selected threshold, LightGBM correctly classified 54 of 70 deaths and 158 of 187 survivors, yielding a sensitivity of 77.1%, specificity of 84.5%, accuracy of 82.5%, positive predictive value of 65.1%, and negative predictive value of 90.8% (Figure 3J). Comparator confusion matrices are shown in Figure S2; RF performed best among the comparator models, whereas LightGBM produced the fewest false‐negative and false‐positive classifications. Subgroup analysis showed acceptable discrimination in most subgroups, although estimates were limited in small strata (Figure S3).

### 3.4 External Validation of the Optimal Model

**FIGURE 4:**
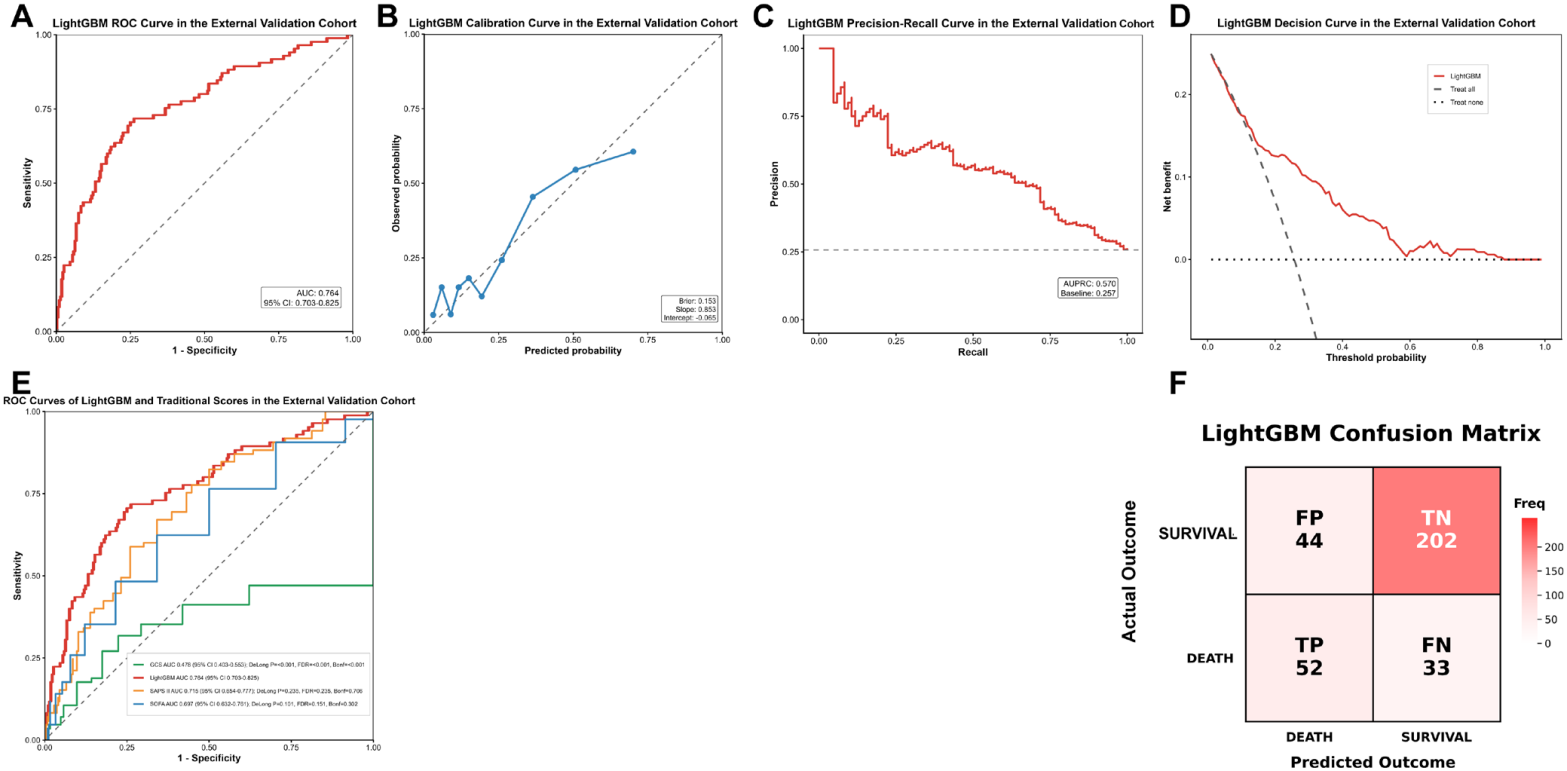
External validation of the model. (A) ROC curve of LightGBM model in the external validation cohort; (B) Calibration curve of LightGBM model in the external validation cohort; (C) PR curve of LightGBM model in the external validation cohort; (D) Decision curve of LightGBM model in the external validation cohort; (E) DeLong's test comparing the discriminative performance of the LightGBM model with traditional clinical scores in the external validation cohort; (F) Confusion matrix of LightGBM in the external validation cohort. AUC, area under the curve; AUPRC, area under the precision–recall curve; AUROC, area under the receiver operating characteristic curve; CI, confidence interval; FN, false negative; FP, false positive; GCS, Glasgow Coma Scale; LightGBM, light gradient boosting machine; ROC, receiver operating characteristic; SAPS II, Simplified Acute Physiology Score II; SOFA, Sequential Organ Failure Assessment; TN, true negative; TP, true positive.

In the external validation cohort, LightGBM showed acceptable external discrimination, with an AUROC of 0.764 (95% CI, 0.703–0.825; Figure 4A). Platt‐calibrated predictions showed acceptable calibration (Brier score, 0.153; calibration intercept, −0.065; calibration slope, 0.853; Figure 4B), and the AUPRC of 0.570 exceeded the event prevalence of 0.257 (Figure 4C). DCA showed greater net benefit than treat‐all and treat‐none strategies across most threshold probabilities (Figure 4D). LightGBM had numerically higher AUROCs than SAPS II, SOFA, and GCS; after DeLong testing and multiple‐comparison correction, only the comparison with GCS remained significant (adjusted *p* < 0.001; Figure 4E). At the selected threshold, the model correctly identified 52 of 85 deaths and 202 of 246 survivors, with a sensitivity of 61.2%, specificity of 82.1%, accuracy of 76.7%, positive predictive value of 54.2%, and negative predictive value of 86.0% (Figure 4F). External‐validation performance metrics of the optimal model are summarized in Table 3.

**TABLE 3:** Key outcome parameters of the LightGBM model in the external validation cohort.

Metric	Value
Threshold	0.320
Youden index	0.433
Accuracy	0.767
Prevalence	0.257
Recall	0.612
Precision	0.542
Specificity	0.821
F1 score	0.575
MCC	0.417
AUROC (95% CI)	0.764 (0.703–0.825)
Brier score	0.153
Calibration intercept	−0.065
Calibration slope	0.853

### 3.5 Sensitivity Analysis

Baseline characteristics of the hematoma‐volume‐complete cohorts are summarized in Table S12. In the first sensitivity analysis, the hematoma‐volume‐augmented LightGBM model achieved an internal out‐of‐fold AUROC of 0.727 (95% CI, 0.656–0.797) and an external AUROC of 0.626 (95% CI, 0.530–0.723). External calibration and net benefit were limited (Brier score, 0.199; calibration intercept, −0.625; calibration slope, 1.493; AUPRC, 0.412; Figure S4). In the second sensitivity analysis, the primary model showed acceptable performance in the external validation subgroup with complete hematoma‐volume data (AUROC, 0.734; 95% CI, 0.666–0.802; Brier score, 0.161; calibration intercept, 0.029; calibration slope, 0.747; AUPRC, 0.508; Figure S5). In the third sensitivity analysis, after re‐including patients with only one or two platelet measurements, the retrained model achieved an internal out‐of‐fold AUROC of 0.827 (95% CI, 0.803–0.851) and an external AUROC of 0.863 (95% CI, 0.819–0.907). This analysis included 1580 model‐development patients and 357 external‐validation patients, with 428 and 91 deaths, respectively. External calibration and decision‐curve findings were supportive (Brier score, 0.129; calibration intercept, 0.338; calibration slope, 1.244; AUPRC, 0.717; Figure S6). Finally, in the sensitivity analysis using only algorithm‐selected predictors, the external validation AUROC decreased from 0.764 (95% CI, 0.703–0.825) for the final expert‐augmented model to 0.717 (95% CI, 0.649–0.782) for the algorithm‐only model (DeLong test, *p* = 0.027; FDR‐adjusted *p* = 0.027; Figure S7). The algorithm‐only model showed a Brier score of 0.168, calibration slope of 1.296, calibration intercept of 0.399, and AUPRC of 0.497.

### 3.6 Model Interpretability Analysis

**FIGURE 5:**
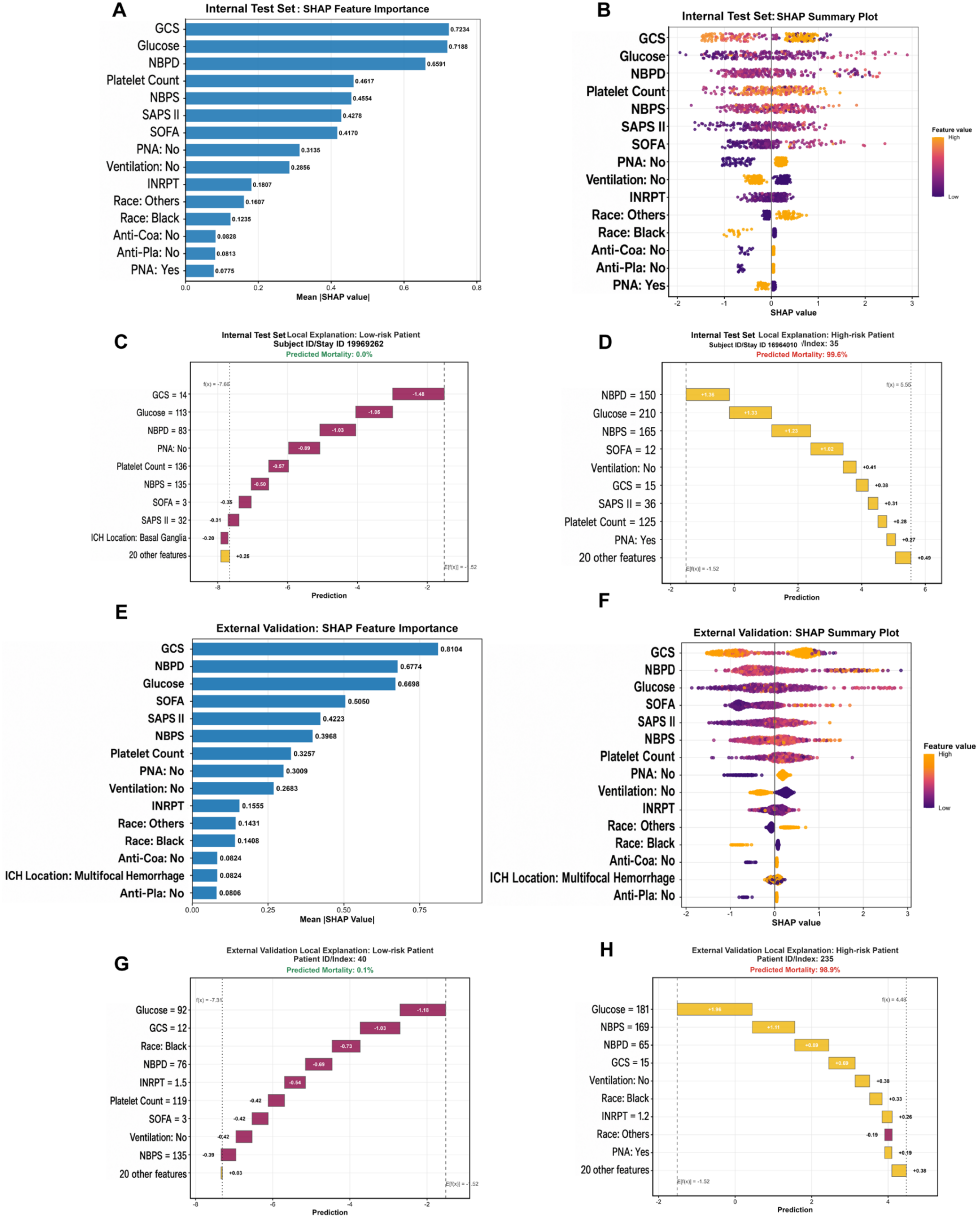
Global and local interpretability analysis of the LightGBM model. (A) Ranking of feature variable importance of the LightGBM model in the internal test set; (B) SHAP beeswarm plot of the LightGBM model in the internal test set; (C) SHAP waterfall plot for local interpretation of a low‐risk patient in the internal test set; (D) SHAP waterfall plot for local interpretation of a high‐risk patient in the internal test set; (E) Ranking of feature variable importance of the LightGBM model in the external validation cohort; (F) SHAP beeswarm plot of the LightGBM model in the external validation cohort; (G) SHAP waterfall plot for local interpretation of a low‐risk patient in the external validation cohort; (H) SHAP waterfall plot for local interpretation of a high‐risk patient in the external validation cohort. Anti‐coa, anticoagulant agent; Anti‐pla, antiplatelet agent; GCS, Glasgow Coma Scale; ICH, intracerebral hemorrhage; INRPT, international normalized ratio of prothrombin time; NBPD, non‐invasive diastolic blood pressure; NBPS, non‐invasive systolic blood pressure; PNA, pneumonia; SAPS II, Simplified Acute Physiology Score II; SHAP, Shapley additive explanations; SOFA, Sequential Organ Failure Assessment.

In the internal test set, GCS, glucose, non‐invasive diastolic blood pressure (NBPD), platelet count, and non‐invasive systolic blood pressure (NBPS) were the top five predictors driving model predictions (Figure 5A). The SHAP summary plot showed broad, bidirectional contributions from GCS, glucose, blood pressure, platelet count, and illness‐severity scores (Figure 5B). Local SHAP analysis illustrated individual predictions. For the selected low‐risk patient in the internal test set (Subject ID/Stay ID: 19969262), several features, particularly GCS, glucose, diastolic blood pressure, absence of pneumonia, and platelet count, shifted the prediction toward a very low mortality probability of 0.0% (Figure 5C). Conversely, in a high‐risk patient (Subject ID/Stay ID: 16964010), diastolic blood pressure, glucose, systolic blood pressure, and SOFA score were the main positive contributors to the predicted mortality probability of 99.6% (Figure 5D).

In the external validation cohort, GCS, NBPD, and glucose remained the leading predictors, demonstrating a feature‐importance pattern broadly consistent with that observed in the internal test set (Figure 5E). The external validation SHAP summary plot confirmed that GCS, glucose, blood pressure variables, platelet count, and illness‐severity scores continued to make substantial contributions to model predictions (Figure 5F). Regarding local interpretability, glucose, GCS, race, diastolic blood pressure, and INRPT contributed to a very low predicted mortality probability of 0.1% (ID: 40; Figure 5G). In contrast, glucose and blood pressure‐related variables were the dominant positive contributors to the predicted mortality probability of 98.9% (ID: 235; Figure 5H).

### 3.7 Clinical Application of the Model

An online calculator was developed based on the final LightGBM model and is available at https://drqiu.shinyapps.io/lightgbm‐mortality‐risk‐calculator/. It is currently being evaluated as a pilot research tool in the Subspecialty of Brain Trauma and Neurocritical Care, Department of Neurosurgery, the First Affiliated Hospital of USTC. The application interface is shown in Figure S8. The preliminary tool is intended for data recording and refinement, not to replace clinical decisions. Prospective multicenter validation is required before routine use.

## 4 Discussion

ICH complicated by thrombocytopenia is a high‐risk neurocritical condition in which impaired hemostasis and bleeding risk may worsen short‐term outcomes [10, 26, 27]. Existing scores such as GCS, SOFA, and SAPS II were not designed specifically for this population and may provide limited individualized risk estimation [28, 29, 30]. Most existing prognostic machine learning models for ICH were developed in general ICH populations rather than in patients with concomitant thrombocytopenia [31]. Differences in case mix, baseline risk, and underlying risk mechanisms may lead to calibration shift when these models are applied to this subgroup, resulting in underestimation or overestimation of the true risk [32, 33]. Therefore, existing ICH prognostic models require dedicated external validation, recalibration, or even model updating with platelet‐related predictors before they can provide reliable prognostic estimates for ICH patients with thrombocytopenia [34].

We developed an interpretable LightGBM model using routinely available variables obtained within 24 h of ICU admission. The model showed good discrimination in the internal test set and acceptable performance in an independent external validation cohort, suggesting its potential as a complementary research‐stage tool for early risk stratification.

Feature selection combined LASSO, Boruta, clinical review, and collinearity assessment to balance statistical relevance with clinical plausibility [35, 36]. Patients with ICH complicated by thrombocytopenia were identified using repeated platelet measurements, which helped reduce the risk of false‐positive classification caused by a single abnormally low platelet count. It should be emphasized that, although the primary cohort included only patients with at least three platelet measurements, all predictors entered into the model were based on the first measurements obtained within 24 h of ICU admission. Furthermore, we performed a sensitivity analysis by re‐including patients who had been excluded because of an insufficient number of platelet measurements. The expanded‐cohort sensitivity analysis showed supportive performance after retraining the model under relaxed platelet‐measurement eligibility criteria.

We attempted to incorporate hematoma location and volume from imaging records and primary diagnoses; however, hematoma volume was frequently unavailable because deidentified CT reports in MIMIC‐IV‐Note often provided qualitative rather than numeric descriptions [37]. Therefore, hematoma volume was excluded from the primary model to avoid substantial missing‐data bias. In sensitivity analyses, a hematoma‐volume‐augmented model retrained in complete cases showed suboptimal performance, likely due to limited sample size and non‐random missingness, whereas the primary model maintained acceptable performance in the hematoma‐volume‐complete external validation subgroup. These findings suggest that the primary model retained useful predictive information despite the absence of hematoma volume.

LightGBM can model non‐linear associations and interactions among routinely available variables, such as neurological status, blood pressure, glucose, platelet count, coagulation status, and systemic illness severity [38]. However, the external validation results require cautious interpretation. Although the model achieved an AUROC of 0.764, its sensitivity at the Youden‐index threshold was 61.2%, and after multiple‐comparison correction it was significantly better than GCS only, but not SOFA or SAPS II. These findings indicate that the incremental discrimination of the model over established generic severity scores may be limited in external validation. Accordingly, the principal value of the model should not be interpreted as universal improvement in predictive accuracy or replacement of existing scores. Rather, it provides subgroup‐specific, probability‐based, and interpretable risk estimation developed specifically for patients with ICH and thrombocytopenia. This tailored risk‐stratification framework may complement generic severity scores by incorporating thrombocytopenia‐related and hemostatic information relevant to this high‐risk population. Prospective multicenter validation, local recalibration, and threshold optimization remain necessary before clinical implementation [38, 39]. In addition, the Youden‐index threshold was used only to standardize performance reporting; clinical thresholds should be tailored to the intended use, balancing sensitivity and specificity.

SHAP analysis improved model transparency by identifying clinically coherent contributors, including GCS, glucose, blood pressure, platelet count, and illness‐severity scores [40, 41, 42, 43]. GCS remains a pivotal indicator for assessing brain injury severity in ICH patients; specifically, those with a GCS < 8 are highly susceptible to severe intracranial hypertension and face a significantly elevated risk of brain herniation [44]. These SHAP findings should be interpreted as contributions to prediction rather than causal effects.

The external validation cohort came from a single tertiary academic teaching hospital in China, where ICU admission criteria, blood pressure management, antithrombotic reversal, ventilation, platelet transfusion, and neurocritical care workflows may differ from those in public ICU databases. Despite these differences, the model showed acceptable external performance, suggesting preliminary transportability. However, mild calibration drift at extreme predicted probabilities indicates that local recalibration and prospective multicenter validation are needed before broader clinical implementation.

## 5 Limitations

Several limitations should be noted. First, the retrospective design, missing data, and inter‐database heterogeneity may introduce selection bias and limit generalizability. Second, important ICH‐specific variables, particularly hematoma volume, prior bleeding history, and other imaging features, were incomplete; therefore, the ICH Score could not be reliably reconstructed and the model was compared only with SOFA, SAPS II, and GCS. Third, residual etiological heterogeneity of ICH may remain because causes such as aneurysm, arteriovenous malformation, postoperative hemorrhage, and congenital coagulation disorders could not be fully identified using structured codes. Fourth, thrombocytopenia etiology was heterogeneous and could reflect infection, medications, liver dysfunction, immune or hematologic disorders, consumptive coagulopathy, or hemorrhage‐related platelet depletion. Definitive etiological stratification was not feasible because adjudicated causes, detailed medication timing, standardized coagulopathy documentation, and clinician‐confirmed thrombocytopenia diagnoses were unavailable. The model should therefore be interpreted as an ICU admission‐time prognostic tool rather than a mechanistic model of thrombocytopenia [16, 45, 46]. Furthermore, excluding patients with ICU stays ≤ 24 h may have underrepresented very early deaths; thus, the model mainly applies to patients remaining in the ICU beyond 24 h. Finally, the online calculator remains a research‐stage tool and should not replace clinical judgment. Future prospective studies with adjudicated etiologies and multicenter validation are needed.

## 6 Conclusion

We developed and externally validated an interpretable LightGBM model for predicting 28‐day all‐cause mortality after ICU admission in ICU patients with ICH and thrombocytopenia. The model showed good internal discrimination, acceptable external discrimination, and clinically coherent SHAP explanations. A web‐based application was developed for research‐stage risk estimation, but further prospective multicenter validation is required before routine clinical use.

## Author Contributions

**Dachang Qiu:** conceptualization, data curation, formal analysis, investigation, methodology, software, validation, visualization, writing – original draft; **Guangwei Li:** methodology, validation; **Ze Wang:** methodology; **Lin Wang:** investigation, visualization; **Lanlan Wang:** visualization, methodology; **Yongfei Dong:** methodology, resources, supervision, writing – review and editing.

## Funding

The authors have nothing to report.

## Ethics Statement

The external validation cohort study was approved by the Medical Research Ethics Committee of the First Affiliated Hospital of the University of Science and Technology of China (Approval No. 2026‐RE‐176), and the requirement for informed consent was waived because of the retrospective nature of the study. The public ICU databases used in this study contain de‐identified data and were approved by their respective institutional review boards. The principal author Dachang Qiu completed the required data‐use training and was granted access to these databases (CITI Program Record ID: 65770488). All procedures were conducted in accordance with the Declaration of Helsinki.

## Conflicts of Interest

The authors declare no conflicts of interest.

## Supporting information


**Table S1:** (A). Missing values of internal cohort. (B) Missing values of external validation cohort.
**Table S2:** Imputation model parameters.
**Table S3:** Clinical rationale for expert‐added baseline variables in the 28‐day mortality prediction model.
**Table S4:** Confidence intervals for calibration metrics.
**Table S5:** Reproducibility details of model development and validation.
**Table S6:** Baseline characteristics of patients.
**Table S7:** Distribution of features in the internal training and test sets.
**Table S8:** Variables selected by the LASSO regression.
**Table S9:** Variables selected by the Boruta.
**Table S10:** VIF Collinearity screening.
**Table S11:** Optimal hyperparameters of the five representative models.
**Table S12:** Key baseline characteristics of patients with available quantitative hematoma volume data.
**Figure S1:** 10‐fold cross‐validation of the five representative models in the internal training set.
**Figure S2:** The confusion matrix of the model on the internal test set; (A) Logistic regression model; (B) multi‐layer perceptron; (C) random forest; (D) support vector machine.
**Figure S3:** Subgroup analysis of the discriminative performance of the optimal model in the internal test set.
**Figure S4:** Performance of the hematoma‐volume‐augmented LightGBM model in the hematoma‐volume‐complete sensitivity analysis. A sensitivity analysis was performed among patients with available quantitative hematoma volume data. A LightGBM model incorporating the 15 admission‐time predictors used in the primary analysis and admission hematoma volume was retrained in the hematoma‐volume‐complete internal cohort and then evaluated in the hematoma‐volume‐complete external validation cohort. (A) Ten‐fold cross‐validation receiver operating characteristic curves in the hematoma‐volume‐complete internal cohort；(B) Receiver operating characteristic curve in the hematoma‐volume‐complete external validation cohort; (C) Calibration curve in the hematoma‐volume‐complete external validation cohort; (D) Precision‐recall curve in the hematoma‐volume‐complete external validation cohort; (E) Decision curve analysis in the hematoma‐volume‐complete external validation cohort.
**Figure S5:** External validation of the primary LightGBM model in the hematoma‐volume‐complete external validation subgroup. The primary LightGBM model trained using the 15 admission‐time predictors was evaluated in the hematoma‐volume‐complete external validation subgroup. The external validation dataset was used directly without incorporating hematoma volume into model prediction. (A) Receiver operating characteristic curve of the LightGBM model, with the AUROC and 95% confidence interval shown. (B) Calibration curve based on Platt‐calibrated predicted probabilities. The dashed diagonal line represents perfect calibration. (C) Precision‐recall curve of the LightGBM model. The dashed horizontal line indicates the event prevalence. (D) Decision curve analysis showing the net benefit of the LightGBM model across threshold probabilities compared with treat‐all and treat‐none strategies. LightGBM, light gradient boosting machine; AUROC, area under the receiver operating characteristic curve.
**Figure S6:** Sensitivity analysis in the expanded thrombocytopenia cohort.
**Figure S7:** Sensitivity analysis using algorithm‐selected predictors only (the eight predictors jointly selected by both LASSO and Boruta).
**Figure S8:** Clinical application of our prediction model. The web application is intended for adult ICU patients with non‐traumatic ICH and thrombocytopenia within 24 h after ICU admission. It provides a Platt‐calibrated probability of 28‐day all‐cause mortality. The reporting threshold was based on the Youden index, but clinical thresholds should be context‐specific. (A) Interactive interface demonstrating how the model predicts low‐risk patients; (B) Interactive interface demonstrating how the model predicts high‐risk patients.

## Data Availability

The data that support the findings of this study are openly available in MIMIC‐III, MIMIC‐IV, eICU‐CRD, and NWICU database at https://physionet.org/content/mimiciv/3.1/, https://physionet.org/content/mimiciii/1.4/, https://eicu‐crd.mit.edu, and https://physionet.org/content/nwicu‐northwestern‐icu/0.1.0/. The web‐based 28‐day mortality prediction tool for patients with ICH and thrombocytopenia is available at this website (https://drqiu.shinyapps.io/lightgbm‐mortality‐risk‐calculator/). The analysis code, a minimal prediction script, and the final trained model object can be made available from the corresponding author upon reasonable request, subject to institutional and data‐use restrictions.
