# Supplementary material for: Development and Validation of an Interpretable Machine Learning‐Based Clinical Prediction Model for Short‐Term Mortality in Intracerebral Hemorrhage With Thrombocytopenia: A Multicenter Study
Source: CNS Neurosci Ther. 2026 Jul 30;32(8):e71067. doi: 10.1002/cns.71067 (PMC13421090; doi:10.1002/cns.71067)
Supplement: Supplementary file 1 — Table S1: (A). Missing values of internal cohort. (B) Missing values of external validation cohort. Table S2: Imputation model parameters. Table S3: Clinical rationale for expert‐added baseline variables in the 28‐day mortality prediction model. Table S4: Confidence intervals for calibration metrics. Table S5: Reproducibility details of model development and validation. Table S6: Baseline characteristics of patients. Table S7: Distribution of features in the internal training and test sets. Table S8: Variables selected by the LASSO regression. Table S9: Variables selected by the Boruta. Table S10: VIF Collinearity screening. Table S11: Optimal hyperparameters of the five representative models. Table S12: Key baseline characteristics of patients with available quantitative hematoma volume data. Figure S1: 10‐fold cross‐validation of the five representative models in the internal training set. Figure S2: The confusion matrix of the model on the internal test set; (A) Logistic regression model; (B) multi‐layer perceptron; (C) random forest; (D) support vector machine. Figure S3: Subgroup analysis of the discriminative performance of the optimal model in the internal test set. Figure S4: Performance of the hematoma‐volume‐augmented LightGBM model in the hematoma‐volume‐complete sensitivity analysis. A sensitivity analysis was performed among patients with available quantitative hematoma volume data. A LightGBM model incorporating the 15 admission‐time predictors used in the primary analysis and admission hematoma volume was retrained in the hematoma‐volume‐complete internal cohort and then evaluated in the hematoma‐volume‐complete external validation cohort. (A) Ten‐fold cross‐validation receiver operating characteristic curves in the hematoma‐volume‐complete internal cohort；(B) Receiver operating characteristic curve in the hematoma‐volume‐complete external validation cohort; (C) Calibration curve in the hematoma‐volume‐complete external validation cohort; (D) Precisio [file CNS-32-e71067-s001.docx]

**Table S1-(A) Missing values of internal cohort**

| **Variable Names** | **Missing(%)** |
| --- | --- |
| Absolute lymphocyte count | 72.5 |
| Hematocrit | 6.8 |
| Hemoglobin | 6.7 |
| RDW | 6.8 |
| RBC | 6.7 |
| WBC | 6.7 |
| Absolute neutrophil count | 72.5 |
| Hemoglobin A1c | 53.5 |
| Albumin | 54.0 |
| Anion gap | 6.1 |
| Calcium total | 6.6 |
| Chloride | 6.1 |
| Globulin | 94.5 |
| Glucose | 6.1 |
| Potassium | 6.1 |
| Protein total | 92.2 |
| Sodium | 5.7 |
| Lactate | 58.3 |
| Pco2 | 51.4 |
| Ph | 50.5 |
| Po2 | 51.4 |
| D-dimer | 98.2 |
| Fibrinogen functional | 87.1 |
| INRPT | 11.3 |
| PT | 11.2 |
| PTT | 11.9 |
| Thrombin | 99.1 |
| Cholesterol hdl | 54.9 |
| Cholesterol ldl calculated | 55.7 |
| Cholesterol total | 54.6 |
| Triglycerides | 51.3 |
| ALT | 27.0 |
| AST | 26.4 |
| Bilirubin direct | 96.1 |
| Bilirubin indirect | 96.2 |
| Bilirubin total | 29.9 |
| Creatinine | 6.2 |
| Urea nitrogen | 6.1 |
| Uric acid | 96.5 |
| Creatine kinase ck | 51.9 |
| Creatine kinase mb isoenzyme | 62.4 |
| Lactate dehydrogenase ld | 60.1 |
| Ntprobnp | 95.3 |
| Troponin t | 81.2 |
| Age | 8.0 |
| Weight | 8.6 |
| Height | 62.6 |
| SOFA | 12.5 |
| APS III | 12.5 |
| SAPS II | 12.5 |
| GCS | 12.8 |
| HR | 8.0 |
| NBPS | 9.3 |
| NBPD | 9.3 |
| ABPS | 64.1 |
| ABPD | 64.1 |
| RR | 8.1 |
| Spo2 | 8.1 |
| Temperature | 9.3 |
| Platelet count | 8.9 |
| ICH Location | 8.0 |
| HTN | 8.0 |
| AKI | 8.0 |
| LC | 8.0 |
| HEP | 8.0 |
| PNA | 8.0 |
| CKD | 8.0 |
| CA | 8.0 |
| T2DM | 8.0 |
| HLD | 8.0 |
| HF | 8.0 |
| MI | 8.0 |
| COPD | 8.0 |
| Anti-coa | 0.0 |
| Anti-pla | 0.0 |
| Death within 28 days | 0.0 |
| Surgery | 8.0 |
| CRRT | 0.0 |
| Ventilation | 0.0 |

**GCS**: Glasgow Coma Scale; **SOFA**: Sequential Organ Failure Assessment; **APS III**: Acute Physiology Score III; **SAPS II**: Simplified Acute Physiology Score II; **HR**: Heart Rate; **RR**: Respiratory Rate; **NBPS**: Non-invasive Blood Pressure Systolic; **NBPD**: Non-invasive Blood Pressure Diastolic; **RDW**: Red Cell Distribution Width; **RBC**: Red Blood Cell; **WBC**: White Blood Cell; **AG**: Anion Gap; **INRPT:** International Normalized Ratio of Prothrombin Time; **PT**: Prothrombin Time; **PTT**: Partial Thromboplastin Time; **ALT**: Alanine Aminotransferase; **AST**: Aspartate Aminotransferase; **TBIL**: Total Bilirubin; **AKI**: Acute Kidney Injury; **HTN**: Hypertension; **LC**: Liver Cirrhosis; **HEP**: Hepatitis; **PNA**: Pneumonia; **CKD**: Chronic Kidney Disease; **CA**: Carcinoma; **T2DM**: Type 2 Diabetes Mellitus; **HLD**: Hyperlipidemia; **HF**: Heart Failure; **MI**: Myocardial Infarction; **COPD**: Chronic Obstructive Pulmonary Disease; **Anti-coa**: Anticoagulation agent; **Anti-pla**: Antiplatelet agent; **CRRT**: Continuous Renal Replacement Therapy;

**Table S1-(B) Missing values of external validation cohort**

| **Variable Names** | **Missing(%)** |
| --- | --- |
| Absolute lymphocyte count | 2.7 |
| Hematocrit | 1.8 |
| Hemoglobin | 1.8 |
| RDW | 2.7 |
| RBC | 2.7 |
| WBC | 2.7 |
| Absolute neutrophil count | 2.7 |
| Albumin | 37.5 |
| Anion gap | 3.9 |
| Calcium total | 3.9 |
| Chloride | 3.9 |
| Glucose | 0.6 |
| Potassium | 3.9 |
| Protein total | 0.6 |
| Sodium | 3.9 |
| Lactate | 7.3 |
| Pco2 | 7.3 |
| PH | 7.3 |
| Po2 | 7.3 |
| D-dimer | 3.3 |
| Fibrinogen functional | 3.3 |
| INRPT | 3.3 |
| PT | 3.3 |
| PTT | 3.3 |
| Thrombin | 90.9 |
| Cholesterol hdl | 0.6 |
| Cholesterol ldl calculated | 0.6 |
| Cholesterol total | 0.6 |
| Triglycerides | 0.6 |
| ALT | 7.7 |
| AST | 7.7 |
| Bilirubin direct | 7.3 |
| Bilirubin indirect | 7.3 |
| Bilirubin total | 7.3 |
| Creatinine | 9.4 |
| Urea nitrogen | 10.3 |
| Uric acid | 8.2 |
| Creatine kinase ck | 14.8 |
| Creatine kinase mb isoenzyme | 17.2 |
| Lactate dehydrogenase ld | 7.9 |
| Ntprobnp | 17.5 |
| Troponin t | 16.3 |
| Age | 0.6 |
| Weight | 3.3 |
| Height | 29.6 |
| SOFA | 3.6 |
| SAPS II | 5.1 |
| APS III | 6.3 |
| GCS | 0.9 |
| HR | 1.8 |
| NBPS | 2.4 |
| NBPD | 2.4 |
| RR | 1.2 |
| Spo2 | 17.8 |
| Temperature | 1.5 |
| Platelet count | 0.6 |
| ICH Location (%) | 0.9 |
| HTN | 0 |
| AKI | 0 |
| LC | 0 |
| HEP | 0 |
| PNA | 0 |
| CKD | 0 |
| CA | 0 |
| T2DM | 0 |
| HLD | 0 |
| HF | 0 |
| MI | 0 |
| COPD | 0 |
| Anti-coa | 0 |
| Anti-pla | 0 |
| Death within 28 days | 0 |
| Surgery | 0 |
| CRRT | 0 |
| Ventilation | 0 |

**GCS**: Glasgow Coma Scale; **SOFA**: Sequential Organ Failure Assessment; **APS III**: Acute Physiology Score III; **SAPS II**: Simplified Acute Physiology Score II; **HR**: Heart Rate; **RR**: Respiratory Rate; **NBPS**: Non-invasive Blood Pressure Systolic; **NBPD**: Non-invasive Blood Pressure Diastolic; **RDW**: Red Cell Distribution Width; **RBC**: Red Blood Cell; **WBC**: White Blood Cell; **AG**: Anion Gap; **INRPT:** International Normalized Ratio of Prothrombin Time; **PT**: Prothrombin Time; **PTT**: Partial Thromboplastin Time; **ALT**: Alanine Aminotransferase; **AST**: Aspartate Aminotransferase; **TBIL**: Total Bilirubin; **AKI**: Acute Kidney Injury; **HTN**: Hypertension; **LC**: Liver Cirrhosis; **HEP**: Hepatitis; **PNA**: Pneumonia; **CKD**: Chronic Kidney Disease; **CA**: Carcinoma; **T2DM**: Type 2 Diabetes Mellitus; **HLD**: Hyperlipidemia; **HF**: Heart Failure; **MI**: Myocardial Infarction; **COPD**: Chronic Obstructive Pulmonary Disease; **Anti-coa**: Anticoagulation agent; **Anti-pla**: Antiplatelet agent; **CRRT**: Continuous Renal Replacement Therapy

**Table S2 imputation model parameters**

| **Component** | **Parameter** | **Value** |
| --- | --- | --- |
| Multiple Imputation (mice package) | Number of imputations | 5 |
| Multiple Imputation (mice package) | method | Random Forest |
| Multiple Imputation (mice package) | ntree (Trees per forest) | 10 (mice package default) |
| Multiple Imputation (mice package) | seed | 123 |
| Imputation workflow | Dataset used to fit imputation model | internal training set only |
| Imputation workflow | Outcome included in imputation model | No |
| Imputation workflow | Application to validation data | Fitted imputation procedure applied unchanged to internal test set and external validation cohort |
| Data checking | Post-imputation checks | Dataset dimensions, remaining missing values, variable ranges, and consistency of model input variables were checked before modeling |

**Table S3. Clinical rationale for expert-added baseline variables in the 28-day mortality prediction model**

| **Expert-added variable** | **Clinical domain** | **Clinical rationale for inclusion** | **Guideline/evidence basis** |
| --- | --- | --- | --- |
| Platelet count | Hemostasis / thrombocytopenia severity | Platelet count is the defining abnormality in this cohort and reflects thrombocytopenia severity and residual primary hemostatic capacity. Lower platelet levels may increase susceptibility to ongoing bleeding, hematoma expansion, and the need for hemostatic intervention, making this variable central to short-term mortality prediction. | ICH guidelines emphasize early correction of hemostatic abnormalities and specific management of thrombocytopenia or antiplatelet-associated ICH. Platelet count is routinely available at presentation. [1,2,4] |
| Non-invasive systolic blood pressure | Hemodynamics / acute blood pressure burden | Elevated systolic blood pressure is common after ICH and is related to hematoma expansion, neurological deterioration, and poor outcome. In thrombocytopenic patients, uncontrolled pressure may further aggravate bleeding risk, while excessive lowering may compromise cerebral perfusion. | AHA/ASA and ESO/EANS guidelines both address acute blood pressure control in ICH, with recommendations centered primarily on systolic blood pressure targets and avoidance of overly aggressive reduction. [1,2] |
| Non-invasive diastolic blood pressure | Hemodynamics / vascular and perfusion status | Diastolic blood pressure complements systolic pressure by reflecting vascular tone, perfusion pressure, and hemodynamic instability. Although guideline targets mainly use systolic pressure, diastolic pressure is routinely recorded and may improve risk estimation by capturing blood-pressure extremes and hemodynamic variation. | Blood pressure is a core component of acute ICH management and is universally available in routine care; inclusion of diastolic pressure is clinically justified as part of baseline hemodynamic assessment. [1,2] |
| Antiplatelet therapy | Medication-related platelet dysfunction | Antiplatelet exposure can impair platelet function independently of platelet count. In thrombocytopenic ICH patients, the combination of reduced platelet number and impaired platelet activity may be particularly relevant to hematoma expansion, procedural bleeding risk, and decisions about platelet transfusion or desmopressin. | ICH guidelines specifically discuss antiplatelet-associated hemorrhage and caution that platelet transfusion decisions depend on clinical context, especially whether emergency neurosurgery is required. [1,2,4] |
| Anticoagulant therapy | Medication-related anticoagulation | Pre-ICH anticoagulant use is a major determinant of early hematoma expansion and poor short-term outcome. It provides information not fully captured by INR/PT because direct oral anticoagulants may not be reliably reflected by routine coagulation tests. | AHA/ASA and ESO/EANS guidelines recommend rapid recognition and reversal of anticoagulant-associated ICH when appropriate, underscoring its prognostic and therapeutic importance. [1,2,4] |
| Intracerebral hemorrhage location | Radiological severity / anatomical risk | Hemorrhage location reflects underlying etiology and determines the risk of neurological deterioration, mass effect, hydrocephalus, and surgical relevance. Infratentorial, deep, lobar, and other locations may carry different risks, making location an important imaging-based predictor of 28-day mortality. | Location is a recognized prognostic element in ICH assessment; infratentorial origin is included in the original ICH Score, a widely used mortality grading scale. [3] |
| Glasgow Coma Scale score | Neurological severity / consciousness | The GCS score captures baseline consciousness and global neurological injury severity. Lower GCS scores indicate more severe brain injury and are strongly associated with early mortality. Because it is simple, routinely recorded, and available at presentation, GCS is clinically indispensable for short-term outcome prediction. | GCS is a core component of established ICH prognostic grading; the original ICH Score assigns increasing points for lower GCS categories and shows a graded relationship with 30-day mortality. [3] |

Abbreviations: GCS, Glasgow Coma Scale; ICH, intracerebral hemorrhage; INR, international normalized ratio; PT, prothrombin time.

**References**

1. Greenberg SM, Ziai WC, Cordonnier C, et al. 2022 Guideline for the Management of Patients With Spontaneous Intracerebral Hemorrhage: A Guideline From the American Heart Association/American Stroke Association. Stroke. 2022;53(7):e282-e361. doi:10.1161/STR.0000000000000407.

2. Steiner T, Al-Shahi Salman R, Beer R, et al. European Stroke Organisation (ESO) and European Association of Neurosurgical Societies (EANS) guideline on stroke due to spontaneous intracerebral haemorrhage. European Stroke Journal. 2025. doi:10.1177/23969873251340815.

3. Hemphill JC 3rd, Bonovich DC, Besmertis L, Manley GT, Johnston SC. The ICH Score: a simple, reliable grading scale for intracerebral hemorrhage. Stroke. 2001;32(4):891-897. doi:10.1161/01.STR.32.4.891.

4. Frontera JA, Lewin JJ 3rd, Rabinstein AA, et al. Guideline for reversal of antithrombotics in intracranial hemorrhage: a statement for healthcare professionals from the Neurocritical Care Society and Society of Critical Care Medicine. Neurocritical Care. 2016;24(1):6-46. doi:10.1007/s12028-015-0222-x.

**Table S4 Confidence intervals for calibration metrics**

| **Model** | **Brier Score (95% CI)** | **Calibration Intercept (95% CI)** | **Calibration Slope (95% CI)** |
| --- | --- | --- | --- |
| LightGBM（Internal test） | 0.126 (0.097-0.158) | -0.456 (-0.782-0.133) | 0.620 (0.463-0.853) |
| LightGBM + Platt（Internal test） | 0.122 (0.097-0.148) | 0.111 (-0.253-0.516) | 1.138 (0.831-1.581) |
| LightGBM + Platt（External validation） | 0.153（0.082-0.223） | -0.065 (-0.207-0.431) | 0.853（0.616-1.184） |

**Table S5. Reproducibility details of model development and validation**

| **Domain** | **Item** | **Details** |
| --- | --- | --- |
| Data source and analysis datasets | Internal cohort and external validation datasets | The modeling script read two preprocessed datasets: Internal_Cohort_Data.xlsx, and External_Cohort_Validation_Data.xlsx. The internal cohort had been split into training and test sets before model development. |
| Prediction time point | Predictor timing | All candidate predictors used in the final model were obtained within 24 hours after ICU admission. |
| Outcome coding | Outcome definition in model code | The outcome was coded as death = 1 and survival = 0. The coding function accepted labels including “death”, “dead”, “died”, “1” as death and “survival”, “survived”, “alive”, “0” as survival. |
| Final predictor set | Admission-time predictors | The final admission-time predictors were race, pneumonia, myocardial infarction, ventilation, glucose, INRPT, SOFA, SAPS II, platelet count, non-invasive systolic blood pressure, non-invasive diastolic blood pressure, anticoagulant use, antiplatelet use, ICH location, and GCS. |
| Categorical variables | Variables treated as categorical | Race, pneumonia, myocardial infarction, ventilation, anticoagulant use, antiplatelet use, and ICH location were treated as categorical variables. |
| Categorical-variable encoding | Dummy coding | Categorical variables were converted to dummy variables using caret::dummyVars() with fullRank = FALSE. Factor levels were defined using the internal training set only. The same factor levels and dummy-variable structure were then applied to the internal test and external validation datasets. |
| Feature alignment | Alignment of model matrices | After dummy coding, the internal test and external validation model matrices were aligned to the internal training matrix. Missing dummy columns were added as 0, extra columns were removed, and column order was matched to the training matrix. |
| Feature scaling | Scaling strategy | Centering and scaling were performed using caret::preProcess() with method = c("center", "scale"). Scaling parameters were estimated from the internal training data and then applied to the internal test and external validation data. |
| Model-specific scaling | Models using scaled or unscaled data | Logistic regression, support vector machine, and multilayer perceptron used the scaled feature matrix. LightGBM and random forest used the unscaled dummy-coded matrix. |
| Near-zero variance handling in cross-validation | Near-zero variance variables | During 10-fold cross-validation, near-zero variance columns were removed for the scaled-model workflow before centering and scaling. |
| Missing-data handling | Missingness screening and imputation | Variables with more than 30% missing values were excluded from model development. Remaining missing values were imputed using random forest multiple imputation. The imputation model was fitted using only the internal training set and without outcome information, and was then applied unchanged to the internal test and external validation cohorts |
| Imputation parameters | Multiple imputation settings | Random forest multiple imputation was performed using the mice package. The number of imputations was 5, the method was random forest, the number of trees per forest was 10, and the imputation seed was 123. |
| Use of imputed datasets in final modeling | Completed datasets | The final modeling code used completed imputed analysis datasets exported as internal training, internal test, and external validation files. Model estimates were derived from these completed analysis datasets rather than from Rubin-pooled regression coefficients. |
| Class-imbalance handling | Resampling and weighting | No oversampling or undersampling procedure was used. SMOTE was not used. For LightGBM, scale_pos_weight was included and was derived from the negative-to-positive outcome ratio in the internal training set unless otherwise specified in the hyperparameter workbook. |
| Candidate models | Models evaluated | Five models were evaluated: LightGBM, random forest, support vector machine, multilayer perceptron, and logistic regression. |
| Hyperparameter parsing | Parameter extraction | A flexible helper function was used to extract parameters from either long-format tables with model, parameter, and value columns or wide-format tables with parameter names as column headers. |
| Hyperparameter search space | Search space reporting | For LightGBM, the search space included learning rates of 0.01, 0.05, and 0.10; num_leaves of 15, 31, and 63; max_depth values of −1, 3, 5, and 7; min_data_in_leaf values of 10, 20, and 30; feature_fraction values of 0.6, 0.8, and 1.0; bagging_fraction values of 0.6, 0.8, and 1.0; bagging_freq values of 0, 1, and 5; lambda_l1 and lambda_l2 values of 0, 0.1, and 1.0; scale_pos_weight values of 1 or the negative-to-positive outcome ratio; and boosting rounds of 100, 150, and 200. The optimal LightGBM parameters were learning_rate = 0.10, num_leaves = 31, max_depth = −1, min_data_in_leaf = 20, feature_fraction = 0.8, bagging_fraction = 1.0, bagging_freq = 0, lambda_l1 = 0, lambda_l2 = 0, scale_pos_weight = the negative-to-positive outcome ratio, and 150 boosting rounds. For random forest, num.trees values of 300, 500, and 1000, mtry values of 2, 3, 4, and 5, and min.node.size values of 1, 3, and 5 were searched, with optimal values of 500, 3, and 1, respectively. For support vector machine, a radial kernel was used, with cost values of 0.1, 1, and 10 and gamma values of 0.01, 0.0417, and 0.1; the optimal values were cost = 1 and gamma = 0.0417. For the multilayer perceptron, hidden units of 5, 10, and 15, weight decay values of 0.001, 0.01, and 0.1, and maximum iterations of 300, 500, and 1000 were evaluated, with optimal values of 10 hidden units, decay = 0.1, and maxit = 500. Logistic regression was fitted using the binomial family with a logit link and therefore did not require additional hyperparameter tuning. |
| LightGBM optimal parameters | Final LightGBM settings | Objective = binary; metric = AUC; boosting type = gbdt; learning rate = 0.1; num leaves = 31; feature fraction = 0.8; min data in leaf = 20; verbosity = −1; number of iterations/boosting rounds = 150. Additional parameters read by the code included max depth, bagging fraction, bagging frequency, L1 regularization, L2 regularization, and scale positive weight. |
| Random forest optimal parameters | Final random forest settings | Random forest was implemented using the ranger package with probability prediction. The reported optimal parameters were number of trees = 500, mtry = 3, and node size/minimum node size = 1. |
| SVM optimal parameters | Final SVM settings | SVM was implemented using the e1071 package as C-classification with probability output. The kernel was standardized in the code; Kernel = radial/RBF. Reported optimal parameters were cost = 1 and gamma = 0.0416666667. |
| MLP optimal parameters | Final MLP settings | MLP was implemented using the nnet package with softmax output. Reported optimal parameters were hidden units = 10, decay = 0.1, and maximum iterations = 500. |
| Logistic regression settings | Final LR settings | Logistic regression was fitted using glm() with binomial family and logit link. |
| Cross-validation | 10-fold internal training cross-validation | Ten-fold cross-validation was performed in the internal training set using caret::createFolds() with k = 10. Out-of-fold predictions and fold-specific AUROCs were generated for all five models. |
| Random seeds | Reproducibility seed | The global random seed was set to 123. In the cross-validation loop, model-specific seeds were set as 123 + fold number. Random forest also used seed = 123 in final model fitting. |
| Final model fitting | Final training | Final models were trained on the full internal training set using the optimized hyperparameters. |
| Internal test evaluation | Independent internal test set | The internal test set was used for final performance evaluation and was not used for feature selection, model training, or hyperparameter tuning. |
| Discrimination metrics | AUROC and confidence intervals | AUROC and 95% confidence intervals were calculated using the pROC package. |
| Precision-recall performance | AUPRC | Precision-recall AUC was calculated using the PRROC package. |
| Calibration metrics | Brier score, intercept, and slope | Brier score was calculated as the mean squared difference between predicted probability and observed outcome. Calibration intercept and slope were estimated by fitting logistic regression with observed outcome as the dependent variable and the logit-transformed predicted probability as the independent variable. |
| Probability truncation for calibration | Safe logit transformation | Predicted probabilities were truncated to the interval 1×10⁻⁶ to 1−1×10⁻⁶ before logit transformation to avoid infinite values. |
| Platt calibration | Calibration workflow | Platt scaling was applied to the final LightGBM model. The saved Platt calibration object was used to transform raw LightGBM probabilities into calibrated probabilities. |
| SHAP analysis | Model interpretation | LightGBM SHAP contributions were extracted using predict(..., type = "contrib"). The bias column was removed before calculating feature-level SHAP values. Mean absolute SHAP values were used for global feature importance. |
| Prediction export | Saved predictions | Predicted probabilities for all five models in the internal test and external validation cohorts were exported as CSV files. |
| Confusion matrix export | Saved confusion matrices | Confusion matrices for each model and cohort were exported as CSV files. |
| Web calculator | Research-stage tool | The web-based prediction tool was developed using the saved final LightGBM model and Platt calibration object. The tool is intended as a research-stage risk-stratification aid and not as an autonomous clinical decision-making system. |
| Code availability | Code and model sharing | The analysis code, a minimal prediction script, and the final trained model object can be made available from the corresponding author upon reasonable request, subject to institutional and data-use restrictions. |
| Software environment | R, packages and their version | Analyses were conducted in R (Version 4.4.1). The modeling code used readxl, dplyr, caret, pROC, ggplot2, lightgbm, ranger, e1071, nnet, tidyr, tibble, purrr, and PRROC. Package Version: readxl 1.4.5; dplyr 1.2.0  ; caret 7.0-1; pROC 1.19.0.1; ggplot2 4.0.2; lightgbm 4.6.0; ranger 0.18.0; e1071 1.7-17; nnet 7.3-19  ; tidyr 1.3.2; purrr 1.2.1; tibble 3.3.1; PRROC 1.4; mice 3.19.0; glmnet 4.1-10; Boruta 9.0.0; car 3.1-5 |

**Table S6 Baseline characteristics of patients**

| **Variable** | **Internal cohort (internal training set+internal test set)**  **N=859（602+257）** | | | **External validation cohort**  **N=331** | | |
| --- | --- | --- | --- | --- | --- | --- |
|  | **Non-death within 28 days**  **N=624** | **Death within 28 days**  **N=235** | ***p*-value** | **Non-death within 28 days**  **N=246** | **Death within 28 days**  **N=85** | ***p*-value** |
| Age | 70.00 (62.00, 80.00) | 72.00 (59.00, 81.00) | 0.941 | 69.00 (63.00, 80.00) | 74.00 (60.00, 83.00) | 0.312 |
| Weight, Kg | 78.40 (64.20, 92.50) | 75.50 (65.00, 90.00) | 0.092 | 74.50 (63.10, 92.00) | 75.90 (66.50, 91.30) | 0.781 |
| SOFA | 5.00 (3.00, 6.00) | 6.00 (4.00, 9.00) | <0.001 | 4.00 (3.00, 7.00) | 5.00 (4.00, 10.00) | 0.002 |
| APS III | 40.50 (30.00, 52.00) | 51.00 (38.00, 76.00) | <0.001 | 40.00 (29.00, 49.00) | 52.50 (40.00, 84.00) | <0.001 |
| SAPS II | 35.00 (28.00, 43.00) | 42.00 (33.00, 55.00) | <0.001 | 35.00 (27.00, 42.00) | 41.50 (34.00, 57.00) | <0.001 |
| GCS | 14.00 (10.00, 15.00) | 14.00 (7.00, 15.00) | 0.176 | 14.00 (11.00, 15.00) | 13.00 (6.00, 15.00) | 0.147 |
| HR, bpm | 82.00 (72.00, 95.00) | 88.00 (74.00, 98.00) | 0.015 | 81.00 (68.00, 93.00) | 86.00 (74.00, 97.00) | 0.090 |
| NBPS, mmHg | 132.00 (120.00, 146.00) | 130.00 (116.00, 145.00) | 0.143 | 133.00 (113.00, 146.00) | 133.00 (117.00, 147.00) | 0.518 |
| NBPD, mmHg | 73.00 (63.00, 84.00) | 69.00 (59.00, 82.00) | 0.037 | 72.00 (63.00, 84.00) | 69.00 (58.00, 89.00) | 0.528 |
| RR, bpm | 19.00 (16.00, 23.00) | 19.00 (15.00, 22.00) | 0.211 | 20.00 (16.00, 24.00) | 19.00 (16.00, 22.00) | 0.356 |
| SPO2, % | 98.00 (96.00, 100.00) | 99.00 (96.00, 100.00) | 0.008 | 97.00 (96.00, 100.00) | 99.00 (97.00, 100.00) | 0.010 |
| Temperature, ℉ | 98.30 (97.80, 98.80) | 98.10 (97.70, 98.90) | 0.174 | 98.10 (97.70, 98.70) | 98.10 (97.80, 99.00) | 0.560 |
| Hematocrit, % | 34.40 (29.70, 39.10) | 34.20 (29.50, 39.10) | 0.863 | 34.90 (29.60, 39.30) | 35.60 (30.70, 39.10) | 0.269 |
| **Variable** | **Internal cohort (internal training set+internal test set)**  **N=859（602+257）** | | | **External validation cohort**  **N=331** | | |
|  | **Non-death within 28 days**  **N=624** | **Death within 28 days**  **N=235** | ***p*-value** | **Non-death within 28 days**  **N=246** | **Death within 28 days**  **N=85** | ***p*-value** |
| Hemoglobin, g/dL | 11.60 (9.80, 13.10) | 11.30 (10.00, 13.10) | 0.887 | 11.60 (9.80, 13.00) | 12.00 (10.50, 13.30) | 0.142 |
| RDW, % | 14.40 (13.40, 16.00) | 14.70 (13.60, 16.00) | 0.209 | 14.50 (13.40, 16.00) | 14.55 (13.60, 15.80) | 0.629 |
| RBC, m/uL | 3.67 (3.10, 4.20) | 3.70 (3.01, 4.27) | 0.811 | 3.64 (3.08, 4.20) | 3.84 (3.26, 4.31) | 0.126 |
| WBC , K/uL | 7.90 (5.70, 11.00) | 10.30 (7.00, 13.30) | <0.001 | 8.30 (5.60, 11.20) | 10.65 (7.20, 13.10) | 0.001 |
| Anion gap , mEq/L | 13.00 (11.00, 15.95) | 15.00 (12.00, 17.00) | <0.001 | 13.00 (11.00, 15.00) | 15.00 (12.00, 17.00) | 0.007 |
| Calcium , mg/dL | 8.60 (8.20, 9.10) | 8.50 (8.00, 8.90) | 0.029 | 8.70 (8.20, 9.20) | 8.55 (8.10, 8.90) | 0.059 |
| Chloride, mEq/L | 104.50 (101.00, 108.00) | 104.00 (100.00, 107.00) | 0.063 | 104.00 (100.00, 107.00) | 104.00 (100.00, 106.00) | 0.437 |
| Glucose , mg/dL | 126.00 (102.00, 158.50) | 134.00 (116.00, 183.00) | <0.001 | 124.00 (99.00, 157.00) | 132.50 (117.00, 183.00) | 0.003 |
| Potassium , mEq/L | 3.90 (3.60, 4.30) | 4.00 (3.50, 4.50) | 0.220 | 3.90 (3.50, 4.30) | 4.00 (3.50, 4.60) | 0.127 |
| Sodium , mEq/L | 139.00 (137.00, 142.00) | 139.00 (136.00, 142.00) | 0.275 | 139.00 (137.00, 142.00) | 139.00 (136.00, 142.00) | 0.340 |
| INRPT | 1.20 (1.10, 1.40) | 1.30 (1.10, 1.50) | 0.012 | 1.20 (1.10, 1.40) | 1.30 (1.10, 1.50) | 0.013 |
| ALT, IU/L | 24.00 (16.00, 43.00) | 24.00 (15.00, 59.00) | 0.403 | 22.00 (16.00, 40.00) | 22.50 (14.00, 63.00) | 0.908 |
| AST, IU/L | 34.00 (23.00, 58.00) | 42.00 (24.00, 78.00) | 0.006 | 34.00 (24.00, 58.00) | 42.50 (24.00, 78.00) | 0.223 |
| **Variable** | **Internal cohort (internal training set+internal test set)**  **N=859（602+257）** | | | **External validation cohort**  **N=331** | | |
|  | **Non-death within 28 days**  **N=624** | **Death within 28 days**  **N=235** | ***p*-value** | **Non-death within 28 days**  **N=246** | **Death within 28 days**  **N=85** | ***p*-value** |
| TBIL, mg/dL | 0.80 (0.50, 1.35) | 0.90 (0.50, 1.50) | 0.054 | 0.80 (0.50, 1.30) | 0.90 (0.50, 1.50) | 0.206 |
| Creatinine, mg/dL | 0.90 (0.70, 1.30) | 1.00 (0.70, 1.60) | 0.018 | 0.90 (0.70, 1.20) | 1.05 (0.80, 1.60) | 0.014 |
| Urea nitrogen, mg/dL | 17.00 (13.00, 27.00) | 19.00 (14.00, 29.00) | 0.013 | 17.00 (13.00, 25.00) | 20.00 (16.00, 28.00) | 0.015 |
| Platelet count, K/uL | 121.00 (91.00, 139.00) | 123.00 (87.00, 139.00) | 0.559 | 124.00 (96.00, 139.50) | 127.75 (95.00, 142.50) | 0.705 |
| **ICH Location** |  |  | 0.011 |  |  | 0.031 |
| basal ganglia | 81.00 (12.98%) | 23.00 (9.79%) |  | 31.00 (12.60%) | 5.00 (5.88%) |  |
| Cerebral lobe | 99.00 (15.87%) | 30.00 (12.77%) |  | 44.00 (17.89%) | 13.00 (15.29%) |  |
| Brainstem | 6.00 (0.96%) | 11.00 (4.68%) |  | 3.00 (1.22%) | 6.00 (7.06%) |  |
| Cerebellum | 44.00 (7.05%) | 13.00 (5.53%) |  | 19.00 (7.72%) | 4.00 (4.71%) |  |
| Ventricle | 56.00 (8.97%) | 26.00 (11.06%) |  | 28.00 (11.38%) | 11.00 (12.94%) |  |
| Multifocal hemorrhage | 6.00 (0.96%) | 1.00 (0.43%) |  | 2.00 (0.81%) | 0.00 (0.00%) |  |
| Others | 332.00 (53.21%) | 131.00 (55.74%) |  | 119.00 (48.37%) | 46.00 (54.12%) |  |
| **Variable** | **Internal cohort (internal training set+internal test set)**  **N=859（602+257）** | | | **External validation cohort**  **N=331** | | |
|  | **Non-death within 28 days**  **N=624** | **Death within 28 days**  **N=235** | ***p*-value** | **Non-death within 28 days**  **N=246** | **Death within 28 days**  **N=85** | ***p*-value** |
| **Sex** |  |  | 0.083 |  |  | 0.003 |
| Female | 239.00 (38.30%) | 75.00 (31.91%) |  | 105.00 (42.68%) | 23.00 (27.06%) |  |
| Male | 385.00 (61.70%) | 160.00 (68.09%) |  | 141.00 (57.32%) | 62.00 (72.94%) |  |
| **Race** |  |  | 0.042 |  |  | 0.002 |
| White | 407.00 (65.22%) | 144.00 (61.28%) |  | 9.00 (3.66%) | 1.00 (1.18%) |  |
| Black | 55.00 (8.81%) | 11.00 (4.68%) |  | 14.00 (5.69%) | 3.00 (3.53%) |  |
| Asian | 26.00 (4.17%) | 12.00 (5.11%) |  | 204.00 (82.93%) | 77.00 (90.58%) |  |
| Others | 136.00 (21.79%) | 68.00 (28.94%) |  | 19.00 (7.72%) | 4.00 (4.71%) |  |
| **HTN** |  |  | 0.696 |  |  | 0.106 |
| No | 304.00 (48.72%) | 118.00 (50.21%) |  | 115.00 (46.75%) | 34.00 (40.00%) |  |
| Yes | 320.00 (51.28%) | 117.00 (49.79%) |  | 131.00 (53.25%) | 51.00 (60.00%) |  |
| **AKI** |  |  | 0.106 |  |  | 0.075 |
| No | 516.00 (82.69%) | 183.00 (77.87%) |  | 203.00 (82.52%) | 61.00 (71.76%) |  |
| **Variable** | **Internal cohort (internal training set+internal test set)**  **N=859（602+257）** | | | **External validation cohort**  **N=331** | | |
|  | **Non-death within 28 days**  **N=624** | **Death within 28 days**  **N=235** | ***p*-value** | **Non-death within 28 days**  **N=246** | **Death within 28 days**  **N=85** | ***p*-value** |
| Yes | 108.00 (17.31%) | 52.00 (22.13%) |  | 43.00 (17.48%) | 24.00 (28.24%) |  |
| **LC** |  |  | 0.552 |  |  | 0.991 |
| No | 571.00 (91.51%) | 212.00 (90.21%) |  | 223.00 (90.65%) | 76.00 (89.41%) |  |
| Yes | 53.00 (8.49%) | 23.00 (9.79%) |  | 23.00 (9.35%) | 9.00 (10.59%) |  |
| **HEP** |  |  | 0.080 |  |  | 0.107 |
| No | 602.00 (96.47%) | 232.00 (98.72%) |  | 234.00 (95.12%) | 84.00 (98.82%) |  |
| Yes | 22.00 (3.53%) | 3.00 (1.28%) |  | 12.00 (4.88%) | 1.00 (1.18%) |  |
| **PNA** |  |  | 0.221 |  |  | 0.110 |
| No | 486.00 (77.88%) | 192.00 (81.70%) |  | 194.00 (78.86%) | 58.00 (68.24%) |  |
| Yes | 138.00 (22.12%) | 43.00 (18.30%) |  | 52.00 (21.14%) | 27.00 (31.76%) |  |
| **CKD** |  |  | 0.627 |  |  | 0.613 |
| No | 553.00 (88.62%) | 211.00 (89.79%) |  | 220.00 (89.43%) | 77.00 (90.59%) |  |
| Yes | 71.00 (11.38%) | 24.00 (10.21%) |  | 26.00 (10.57%) | 8.00 (9.41%) |  |
| **Variable** | **Internal cohort (internal training set+internal test set)**  **N=859（602+257）** | | | **External validation cohort**  **N=331** | | |
|  | **Non-death within 28 days**  **N=624** | **Death within 28 days**  **N=235** | ***p*-value** | **Non-death within 28 days**  **N=246** | **Death within 28 days**  **N=85** | ***p*-value** |
| **CA** |  |  | 0.115 |  |  | 0.369 |
| No | 544.00 (87.18%) | 214.00 (91.06%) |  | 213.00 (86.59%) | 76.00 (89.41%) |  |
| Yes | 80.00 (12.82%) | 21.00 (8.94%) |  | 33.00 (13.41%) | 9.00 (10.59%) |  |
| **T2DM** |  |  | 0.993 |  |  | 0.845 |
| No | 507.00 (81.25%) | 191.00 (81.28%) |  | 190.00 (77.24%) | 65.00 (76.47%) |  |
| Yes | 117.00 (18.75%) | 44.00 (18.72%) |  | 56.00 (22.76%) | 20.00 (23.53%) |  |
| **HLD** |  |  | 0.027 |  |  | 0.072 |
| No | 465.00 (74.52%) | 192.00 (81.70%) |  | 168.00 (68.29%) | 65.00 (76.47%) |  |
| Yes | 159.00 (25.48%) | 43.00 (18.30%) |  | 78.00 (31.71%) | 20.00 (23.53%) |  |
| **HF** |  |  | 0.837 |  |  | 0.830 |
| No | 558.00 (89.42%) | 209.00 (88.94%) |  | 216.00 (87.80%) | 73.00 (85.88%) |  |
| Yes | 66.00 (10.58%) | 26.00 (11.06%) |  | 30.00 (12.20%) | 12.00 (14.12%) |  |
| **MI** |  |  | 0.029 |  |  | 0.251 |
| **Variable** | **Internal cohort (internal training set+internal test set)**  **N=859（602+257）** | | | **External validation cohort**  **N=331** | | |
|  | **Non-death within 28 days**  **N=624** | **Death within 28 days**  **N=235** | ***p*-value** | **Non-death within 28 days**  **N=246** | **Death within 28 days**  **N=85** | ***p*-value** |
| No | 610.00 (97.76%) | 223.00 (94.89%) |  | 239.00 (97.15%) | 80.00 (94.12%) |  |
| Yes | 14.00 (2.24%) | 12.00 (5.11%) |  | 7.00 (2.85%) | 5.00 (5.88%) |  |
| **COPD** |  |  | 0.297 |  |  | 0.364 |
| No | 586.00 (93.91%) | 225.00 (95.74%) |  | 232.00 (94.31%) | 82.00 (96.47%) |  |
| Yes | 38.00 (6.09%) | 10.00 (4.26%) |  | 14.00 (5.69%) | 3.00 (3.53%) |  |
| **Anti-coa** |  |  | 0.166 |  |  | 0.542 |
| No | 572.00 (91.67%) | 222.00 (94.47%) |  | 228.00 (92.68%) | 80.00 (94.12%) |  |
| Yes | 52.00 (8.33%) | 13.00 (5.53%) |  | 18.00 (7.32%) | 5.00 (5.88%) |  |
| **Anti-pla** |  |  | 0.010 |  |  | 0.170 |
| No | 579.00 (92.79%) | 229.00 (97.45%) |  | 224.00 (91.06%) | 78.00 (91.76%) |  |
| Yes | 45.00 (7.21%) | 6.00 (2.55%) |  | 22.00 (8.94%) | 7.00 (8.24%) |  |
| **Surgery** |  |  | 0.990 |  |  | 0.251 |
| No | 592.00 (94.87%) | 223.00 (94.89%) |  | 235.00 (95.53%) | 78.00 (91.76%) |  |
| **Variable** | **Internal cohort (internal training set+internal test set)**  **N=859（602+257）** | | | **External validation cohort**  **N=331** | | |
|  | **Non-death within 28 days**  **N=624** | **Death within 28 days**  **N=235** | ***p*-value** | **Non-death within 28 days**  **N=246** | **Death within 28 days**  **N=85** | ***p*-value** |
| Yes | 32.00 (5.13%) | 12.00 (5.11%) |  | 11.00 (4.47%) | 7.00 (8.24%) |  |
| **CRRT** |  |  | 0.002 |  |  | 0.117 |
| No | 608.00 (97.44%) | 218.00 (92.77%) |  | 239.00 (97.15%) | 79.00 (92.94%) |  |
| Yes | 16.00 (2.56%) | 17.00 (7.23%) |  | 7.00 (2.85%) | 6.00 (7.06%) |  |
| **Ventilation** |  |  | <0.001 |  |  | 0.018 |
| No | 293.00 (46.96%) | 62.00 (26.38%) |  | 106.00 (43.09%) | 20.00 (23.53%) |  |
| Yes | 331.00 (53.04%) | 173.00 (73.62%) |  | 140.00 (56.91%) | 65.00 (76.47%) |  |

**Note:** Baseline comparisons were based on the completed imputed analysis datasets**; GCS**: Glasgow Coma Scale; **SOFA**: Sequential Organ Failure Assessment; **APS III**: Acute Physiology Score III; **SAPS II**: Simplified Acute Physiology Score II; **HR**: Heart Rate; **RR**: Respiratory Rate; **NBPS**: Non-invasive Blood Pressure Systolic; **NBPD**: Non-invasive Blood Pressure Diastolic; **RDW**: Red Cell Distribution Width; **RBC**: Red Blood Cell; **WBC**: White Blood Cell; **AG**: Anion Gap; **INRPT:** International Normalized Ratio of Prothrombin Time; **PT**: Prothrombin Time; **PTT**: Partial Thromboplastin Time; **ALT**: Alanine Aminotransferase; **AST**: Aspartate Aminotransferase; **TBIL**: Total Bilirubin; **AKI**: Acute Kidney Injury; **HTN**: Hypertension; **LC**: Liver Cirrhosis; **HEP**: Hepatitis; **PNA**: Pneumonia; **CKD**: Chronic Kidney Disease; **CA**: Carcinoma; **T2DM**: Type 2 Diabetes Mellitus; **HLD**: Hyperlipidemia; **HF**: Heart Failure; **MI**: Myocardial Infarction; **COPD**: Chronic Obstructive Pulmonary Disease; **Anti-coa**: Anticoagulation agent; **Anti-pla**: Antiplatelet agent; **CRRT**: Continuous Renal Replacement Therapy

**Table S7 Distribution of features in the internal training and test sets**

| **Variable Names** | **Level** | **Internal training set**  **N=602** | **Internal test set**  **N=257** | ***p*-value** | **SMD** |
| --- | --- | --- | --- | --- | --- |
| Glucose |  | 132 (107-162) | 125 (103-159) | 0.054 | 0.155 |
| INRPT |  | 1.2 (1.1-1.5) | 1.2 (1.1-1.4) | 0.100 | 0.135 |
| SOFA |  | 5 (3-7) | 5 (3-7) | 0.163 | 0.105 |
| SAPS II |  | 36 (29-46) | 35 (29-46) | 0.280 | 0.081 |
| Platelet count |  | 118.75 (88-138) | 126 (96-139) | 0.153 | 0.107 |
| NBPS |  | 132 (119-146) | 132 (118-146) | 0.465 | 0.055 |
| NBPD |  | 71.5 (63-84) | 73 (62-85) | 0.664 | 0.032 |
| GCS |  | 14 (9.25-15) | 14 (10-15) | 0.162 | 0.105 |
| Race (%) | White | 398 (66.11) | 153 (59.53) | 0.205 | 0.157 |
| Race (%) | Black | 46 (7.64) | 20 (7.78) |  |  |
| Race (%) | Asian | 27 (4.49) | 11 (4.28) |  |  |
| Race(%) | Others | 131 (21.76) | 73 (28.40) |  |  |
| PNA(%) | No | 477 (79.24) | 201 (78.21) | 0.806 | 0.025 |
| PNA(%) | Yes | 125 (20.76) | 56 (21.79) |  |  |
| MI (%) | No | 582 (96.68) | 251 (97.67) | 0.578 | 0.060 |
| MI(%) | Yes | 20 (3.32) | 6 (2.33) |  |  |
| Ventilation (%) | No | 252 (41.86) | 103 (40.08) | 0.682 | 0.036 |
| Ventilation (%) | Yes | 350 (58.14) | 154 (59.92) |  |  |
| Anti-coa (%) | No | 555 (92.19) | 239 (93.00) | 0.790 | 0.031 |
| Anti-coa (%) | Yes | 47 (7.81) | 18 (7.00) |  |  |
| Anti-pla (%) | No | 567 (94.19) | 241 (93.77) | 0.939 | 0.017 |
| Anti-pla (%) | Yes | 35 (5.81) | 16 (6.23) |  |  |
| ICH Location (%) | Basal ganglia | 73 (12.13) | 31 (12.06) | 0.678 | 0.170 |
| ICH Location (%) | Cerebral lobe | 86 (14.29) | 43 (16.73) |  |  |
| ICH Location (%) | Brainstem | 12 (1.99) | 5 (1.95) |  |  |
| ICH Location (%) | Cerebellum | 41 (6.81) | 16 (6.23) |  |  |
| ICH Location (%) | Ventricle | 56 (9.30) | 26 (10.12) |  |  |
| ICH Location (%) | Multifocal hemorrhage | 7 (1.16) | 0 (0.00) |  |  |
| ICH Location (%) | Others | 327 (54.32) | 136 (52.92) |  |  |
| Death within 28d (%) | Death | 165 (27.41) | 70 (27.24) | 1.000 | 0.004 |
| Death within 28d (%) | Survival | 437 (72.59) | 187 (72.76) |  |  |

**GCS**: Glasgow Coma Scale; **SOFA**: Sequential Organ Failure Assessment;**SAPS II**: Simplified Acute Physiology Score II; **NBPS**: Non-invasive Blood Pressure Systolic; **NBPD**: Non-invasive Blood Pressure Diastolic; **PNA**: Pneumonia; **MI**: Myocardial Infarction; **INRPT:** International Normalized Ratio of Prothrombin Time; **Anti-coa**: Anticoagulation agent; **Anti-pla**: Antiplatelet agent; **CRRT**: Continuous Renal Replacement Therapy.

**Table S8 Variables selected by the LASSO regression**

| **Feature** | **Coefficient** |
| --- | --- |
| Race | 0.456745115306383 |
| PNA | -0.101325141 |
| MI | 0.509898755450058 |
| Anti-coa | -0.053986361 |
| Anti-pla | -0.096963907 |
| Ventilation | 0.268693040940197 |
| Glucose | 0.00055505446993498 |
| INRPT | 0.00803815714921114 |
| ALT | 0.000163266647686448 |
| TBIL | 0.00261022521165415 |
| SOFA | 0.0247025959182884 |
| SAPS II | 0.0120319869648508 |
| Temperature | -0.042242569 |

**SOFA**: Sequential Organ Failure Assessment;**SAPS II**: Simplified Acute Physiology Score II; **NBPS**: Non-invasive Blood Pressure Systolic; **NBPD**: Non-invasive Blood Pressure Diastolic; **PNA**: Pneumonia; **MI**: Myocardial Infarction; **INRPT:** International Normalized Ratio of Prothrombin Time; **Anti-coa**: Anticoagulation agent; **Anti-pla**: Antiplatelet agent; **ALT**: Alanine Aminotransferase; **TBIL**: Total Bilirubin. LASSO regression retained 13 variables using the λ1se criterion.

**Table S9 Variables selected by the Boruta**

| **MeanImp** | **MedianImp** | **MinImp** | **MaxImp** | **NormHits** | **Decision** | **Feature** |
| --- | --- | --- | --- | --- | --- | --- |
| -0.004532499 | 0.0381837733275462 | -1.00267279 | 0.999680523149158 | 0 | Rejected | ICH Location |
| 0.128340741582927 | -0.103621985 | -1.055729146 | 2.18767607533814 | 0 | Rejected | Sex |
| 3.38048882502202 | 3.47095729486918 | 1.09342413726091 | 6.1820898965548 | 0.777777777777778 | Confirmed | Race |
| 0.494880849914548 | 0.532601609075197 | -1.151604508 | 2.05967335679837 | 0 | Rejected | HTN |
| -0.104031641 | 0.304509934888813 | -1.650817029 | 1.29612147282715 | 0 | Rejected | AKI |
| 0.280579800283921 | 0.468660111018573 | -1.513586013 | 1.71573717434127 | 0 | Rejected | LC |
| 0.00114797745501569 | 0.0263064431466937 | -1.343617859 | 1.00250941423417 | 0 | Rejected | HEP |
| 2.4393318405958 | 2.53344759860302 | -0.343466959 | 4.88563063416227 | 0.585858585858586 | Confirmed | PNA |
| 0.205786235228787 | 0.847297100913594 | -1.567331538 | 1.49922605326792 | 0 | Rejected | CKD |
| -1.049508931 | -1.372177687 | -2.347436004 | 0.841959318194514 | 0 | Rejected | CA |
| 0.019657231230432 | 0.122718629580556 | -2.885126607 | 1.69224068553249 | 0 | Rejected | T2DM |
| 0.318432956060776 | 0.267432147749954 | -1.088794428 | 1.39373984872339 | 0 | Rejected | HLD |
| 0.242775037673676 | 0.0838328916933496 | -1.302730968 | 2.11903064344555 | 0 | Rejected | HF |
| 5.8982457941902 | 5.8682256884255 | 2.63667577127572 | 8.61542184278206 | 0.97979797979798 | Confirmed | MI |
| 0.172219799204169 | 0.415555142280065 | -1.839104068 | 1.47604756347914 | 0 | Rejected | COPD |
| 0.72086265426498 | 0.897640695246002 | -1.305269613 | 2.13093602477757 | 0.0101010101010101 | Rejected | Anti- coa |
| 0.520753254861356 | 0.456939371760798 | -1.885074855 | 1.90645452904612 | 0.0101010101010101 | Rejected | Anti-pla |
| 0.0568581592473014 | -0.007511853 | -0.927928263 | 1.26296124126979 | 0 | Rejected | Surgery |
| 0.458198333959933 | 0.546517192913022 | -1.729409793 | 2.16447111896377 | 0.0101010101010101 | Rejected | CRRT |
| 2.62653648178614 | 2.66273491422829 | -0.16418976 | 4.83294050616019 | 0.606060606060606 | Confirmed | Ventilation |
| 1.3928739335989 | 1.6173841638987 | -0.700212959 | 2.49889755733356 | 0.0101010101010101 | Rejected | Hematocrit |
| 1.48565211038663 | 1.57530270522442 | -0.912148791 | 3.97316388668536 | 0.272727272727273 | Rejected | Hemoglobin |
| 0.414172327586604 | 0.565142405777174 | -1.117091062 | 1.46248354186133 | 0 | Rejected | RDW |
| 1.12675867650105 | 1.12640995100943 | -0.958973612 | 3.13845555098062 | 0.0101010101010101 | Rejected | RBC |
| 5.34653487656129 | 5.23801351529838 | 1.56398046090164 | 9.00399103636235 | 0.939393939393939 | Confirmed | WBC |
| 2.37582063294017 | 2.35461516308927 | 0.28747204658337 | 4.98386051420316 | 0.535353535353535 | Confirmed | AG |
| 1.61905195458199 | 1.58634994328049 | -1.077766047 | 3.73589741790594 | 0.282828282828283 | Rejected | Calcium |
| 1.82446664213031 | 1.76209440789141 | -1.390868754 | 4.9801308166319 | 0.303030303030303 | Rejected | Chloride |
| 2.57162619352269 | 2.56134498891593 | 0.213205746482274 | 5.37648204270643 | 0.575757575757576 | Confirmed | Glucose |
| 0.0708423436153631 | -0.170872715 | -1.036623892 | 1.27974949186742 | 0 | Rejected | Potassium |
| 1.96211821613863 | 1.9844662421037 | -0.517221917 | 4.42239942704064 | 0.363636363636364 | Rejected | Sodium |
| 2.29815882066877 | 2.46825041573042 | -0.167513715 | 4.49166645162769 | 0.535353535353535 | Confirmed | INRPT |
| 1.52045859790887 | 1.54925053043833 | -0.759923133 | 3.90076987747828 | 0.121212121212121 | Rejected | ALT |
| 1.58002629557804 | 1.62497523357419 | -0.150124893 | 3.30756466694445 | 0.0202020202020202 | Rejected | AST |
| 1.03810076995903 | 1.27683475207873 | -1.506353929 | 3.02542267561694 | 0.0101010101010101 | Rejected | TBIL |
| 0.850574032874719 | 0.636624595180073 | -0.170224729 | 1.92183611966203 | 0 | Rejected | Creatinine |
| 0.602600671007987 | 0.446381334132363 | -0.357332008 | 2.17034148589263 | 0 | Rejected | Urea nitrogen |
| 0.590395402723799 | 0.36441253943586 | -1.086658735 | 1.86356229173871 | 0 | Rejected | Age |
| 0.848144729413552 | 0.940342306203625 | -0.659864749 | 1.6293765483244 | 0 | Rejected | Weight |
| 3.79893414581463 | 3.82228738078374 | 0.810438998455077 | 6.38263329106707 | 0.888888888888889 | Confirmed | SOFA |
| 3.67930362927355 | 3.74499125200144 | 1.17603484768934 | 5.97857736339748 | 0.828282828282828 | Confirmed | APS III |
| 4.33276802417415 | 4.19276819542931 | 1.73890963472041 | 7.37075188329429 | 0.929292929292929 | Confirmed | SAPS II |
| 3.62284729093247 | 3.68367950677162 | 1.46131366100185 | 5.86491880124645 | 0.838383838383838 | Confirmed | GCS |
| 0.172379006812461 | -0.067278822 | -1.402970574 | 1.24515025627633 | 0 | Rejected | HR |
| 1.69038428567948 | 1.72705462382988 | -0.58970413 | 3.98967292907341 | 0.181818181818182 | Rejected | NBPS |
| 1.23456159113083 | 1.06962706183094 | -1.490023814 | 3.48655541638305 | 0.0505050505050505 | Rejected | NBPD |
| 0.105304240995235 | 0.208876162254969 | -2.173009159 | 1.43668703748378 | 0 | Rejected | RR |
| 0.620833833675377 | 0.425030429582984 | -1.104308938 | 2.22514989948076 | 0 | Rejected | SPO2 |
| 1.22587211998453 | 1.20076139379559 | -0.697246224 | 3.19269088729359 | 0.0202020202020202 | Rejected | Temperature |
| 0.40211745793647 | 0.704054474069835 | -1.110367658 | 1.57915054972637 | 0 | Rejected | Platelet count |

**GCS**: Glasgow Coma Scale; **SOFA**: Sequential Organ Failure Assessment; **APS III**: Acute Physiology Score III; **SAPS II**: Simplified Acute Physiology Score II; **HR**: Heart Rate; **RR**: Respiratory Rate; **NBPS**: Non-invasive Blood Pressure Systolic; **NBPD**: Non-invasive Blood Pressure Diastolic; **RDW**: Red Cell Distribution Width; **RBC**: Red Blood Cell; **WBC**: White Blood Cell; **AG**: Anion Gap; **INRPT:** International Normalized Ratio of Prothrombin Time; **PT**: Prothrombin Time; **PTT**: Partial Thromboplastin Time; **ALT**: Alanine Aminotransferase; **AST**: Aspartate Aminotransferase; **TBIL**: Total Bilirubin; **AKI**: Acute Kidney Injury; **HTN**: Hypertension; **LC**: Liver Cirrhosis; **HEP**: Hepatitis; **PNA**: Pneumonia; **CKD**: Chronic Kidney Disease; **CA**: Carcinoma; **T2DM**: Type 2 Diabetes Mellitus; **HLD**: Hyperlipidemia; **HF**: Heart Failure; **MI**: Myocardial Infarction; **COPD**: Chronic Obstructive Pulmonary Disease; **Anti-coa**: Anticoagulation agent; **Anti-pla**: Antiplatelet agent; **CRRT**: Continuous Renal Replacement Therapy;

**Table S10 VIF Collinearity screening**

| **Feature** | **VIF** |
| --- | --- |
| Race | 1.14722846724569 |
| PNA | 1.10092278324917 |
| MI | 1.11379967728208 |
| Ventilation | 1.12988548792827 |
| Glucose | 1.04951978642753 |
| INRPT | 1.25014568076592 |
| SOFA | 2.12770030708933 |
| SAPS II | 1.89980530389478 |
| Platelet count | 1.37003848409869 |
| NBPS | 1.46557665091228 |
| NBPD | 1.41798266349186 |
| Anti-coa | 1.09758988917788 |
| Anti-pla | 1.03708912894797 |
| ICH Location | 1.28552267172738 |
| GCS | 1.4917226805469 |

**SOFA**: Sequential Organ Failure Assessment; **SAPS II**: simplified acute physiology score II; **GCS:** Glasgow Coma Scale; **INRPT:** International Normalized Ratio Prothrombin Time; **PNA**: pneumonia; **MI**: myocardial infarction; **NBPS**: non-invasive systolic blood pressure; **NBPD**: non-invasive diastolic blood pressure; **Anti-coa**: anticoagulant; **Anti-pla**: antiplatelet; **Collinearity screening threshold：5**

**Table S11** **Optimal hyperparameters of the five representative models**

| **Model** | **Parameter** | **Value** |
| --- | --- | --- |
| LightGBM | Boosting type | gbdt |
| LightGBM | Learning rate | 0.1 |
| LightGBM | Num leaves | 31 |
| LightGBM | Feature fraction | 0.8 |
| LightGBM | Min data in leaf | 20 |
| LightGBM | verbosity | -1 |
| LightGBM | Num iterations | 150 |
| RF | Ntree | 500 |
| RF | Mtry | 3 |
| RF | Nodesize | 1 |
| SVM | Kernel | radial/RBF |
| SVM | Cost | 1 |
| SVM | Gamma | 0.0416666666666667 |
| MLP | Size (hidden units) | 10 |
| MLP | Decay | 0.1 |
| MLP | Maxit | 500 |
| LR | Family | binomial |
| LR | Link | logit |

**RF**：Random Forest；**LightGBM**：Light Gradient Boosting Machine；**SVM**：Support Vector Machine；**MLP**：Multilayer Perceptron；

**Table S12 Key baseline characteristics of patients with available quantitative hematoma volume data**

| **Variable** | **Internal cohort**  **N=207** | | | **external validation cohort**  **N=190** | | |
| --- | --- | --- | --- | --- | --- | --- |
|  | **Non-death within 28 days**  **N=136** | **Death within 28 days**  **N=71** | ***p*-value** | **Non-death within 28 days**  **N=142** | **Death within 28 days**  **N=48** | ***p*-value** |
| SOFA | 5.00 (3.00 - 7.00) | 5.00 (3.00 - 9.00) | 0.024 | 2.00 (1.00 - 4.00) | 4.00 (2.00 - 6.00) | <0.001 |
| SAPS II | 33.00 (28.00 - 42.00) | 38.00 (31.00 - 48.00) | 0.007 | 31.00 (24.00 - 36.00) | 37.00 (31.50 - 44.00) | <0.001 |
| GCS | 14.00 (10.50 - 15.00) | 15.00 (10.00 - 15.00) | 0.099 | 14.00 (12.00 - 15.00) | 14.50 (11.00 - 15.00) | 0.956 |
| NBPS, mmHg | 134.50 (120.00 - 146.00) | 136.00 (119.00 - 150.00) | 0.542 | 135.00 (121.00 - 150.00) | 137.00 (124.50 - 155.50) | 0.438 |
| NBPD, mmHg | 74.50 (64.00 - 84.50) | 69.00 (59.00 - 88.00) | 0.274 | 74.50 (64.00 - 86.00) | 76.50 (62.50 - 86.00) | 0.848 |
| INRPT | 1.20 (1.10 - 1.40) | 1.20 (1.10 - 1.40) | 0.798 | 1.10 (1.10 - 1.20) | 1.20 (1.10 - 1.40) | 0.003 |
| Platelet count, K/uL | 121.00 (100.00 - 137.00) | 125.00 (82.50 - 139.00) | 0.557 | 117 .00(96.00-142.00) | 106.00 (87.00 - 142.00) | 0.014 |
| ICH volume | 33.82 (8.57 - 63.81) | 50.00 (38.76 - 70.00) | <0.001 | 50.00 (16.70 - 70.00) | 50.00 (15.56 - 70.00) | 0.658 |
| Glucose , mg/dL | 120.50 (102.00 - 149.00) | 149.00 (119.00 - 186.00) | <0.001 | 124.50 (104.00 - 153.00) | 153.00 (126.50 - 168.00) | 0.002 |
| **ICH Location** |  |  | 0.305 |  |  | 0.753 |
| basal ganglia | 5.15% (7.00 / 136.00) | 7.04% (5.00 / 71.00) |  | 7.04% (10.00 / 142.00) | 6.25% (3.00 / 48.00) |  |
| Cerebral lobe | 16.91% (23.00 / 136.00) | 15.49% (11.00 / 71.00) |  | 17.61% (25.00 / 142.00) | 14.58% (7.00 / 48.00) |  |
| Brainstem | 0.00% (0.00 / 136.00) | 4.23% (3.00 / 71.00) |  | 1.41% (2.00 / 142.00) | 2.08% (1.00 / 48.00) |  |
| Cerebellum | 6.62% (9.00 / 136.00) | 4.23% (3.00 / 71.00) |  | 6.34% (9.00 / 142.00) | 2.08% (1.00 / 48.00) |  |
| Ventricle | 13.24% (18.00 / 136.00) | 15.49% (11.00 / 71.00) |  | 11.27% (16.00 / 142.00) | 18.75% (9.00 / 48.00) |  |
| Multifocal hemorrhage | 0.74% (1.00 / 136.00) | 1.41% (1.00 / 71.00) |  | 0.70% (1.00 / 142.00) | 0.00% (0.00 / 48.00) |  |
| Others | 57.35% (78.00 / 136.00) | 52.11% (37.00 / 71.00) |  | 55.63% (79.00 / 142.00) | 56.25% (27.00 / 48.00) |  |
| **Race** |  |  | 0.028 |  |  | 0.285 |
| White | 64.71% (88.00 / 136.00) | 47.89% (34.00 / 71.00) |  | 2.82% (4.00 / 142.00) | 0.00% (0.00 / 48.00) |  |
| Black | 8.09% (11.00 / 136.00) | 4.23% (3.00 / 71.00) |  | 3.52% (5.00 / 142.00) | 4.17% (2.00 / 48.00) |  |
| Asian | 2.94% (4.00 / 136.00) | 5.63% (4.00 / 71.00) |  | 86.62% (123.00 / 142.00) | 81.25% (39.00 / 48.00) |  |
| Others | 24.26% (33.00 / 136.00) | 42.25% (30.00 / 71.00) |  | 7.04% (10.00 / 142.00) | 14.58% (7.00 / 48.00) |  |
| **MI** |  |  | <0.001 |  |  | 0.622 |
| No | 99.26% (135.00 / 136.00) | 88.73% (63.00 / 71.00) |  | 96.48% (137.00 / 142.00) | 97.92% (47.00 / 48.00) |  |
| Yes | 0.74% (1.00 / 136.00) | 11.27% (8.00 / 71.00) |  | 3.52% (5.00 / 142.00) | 2.08% (1.00 / 48.00) |  |
| **PNA** |  |  | 0.863 |  |  | 0.008 |
| No | 75.74% (103.00 / 136.00) | 74.65% (53.00 / 71.00) |  | 84.51% (120.00 / 142.00) | 66.67% (32.00 / 48.00) |  |
| Yes | 24.26% (33.00 / 136.00) | 25.35% (18.00 / 71.00) |  | 15.49% (22.00 / 142.00) | 33.33% (16.00 / 48.00) |  |
| **Anti-coa** |  |  | 0.027 |  |  | 0.271 |
| No | 90.44% (123.00 / 136.00) | 98.59% (70.00 / 71.00) |  | 95.77% (136.00 / 142.00) | 91.67% (44.00 / 48.00) |  |
| Yes | 9.56% (13.00 / 136.00) | 1.41% (1.00 / 71.00) |  | 4.23% (6.00 / 142.00) | 8.33% (4.00 / 48.00) |  |
| **Anti-pla** |  |  | 0.950 |  |  | 0.636 |
| No | 95.59% (130.00 / 136.00) | 95.77% (68.00 / 71.00) |  | 93.66% (133.00 / 142.00) | 91.67% (44.00 / 48.00) |  |
| Yes | 4.41% (6.00 / 136.00) | 4.23% (3.00 / 71.00) |  | 6.34% (9.00 / 142.00) | 8.33% (4.00 / 48.00) |  |
| **Ventilation** |  |  | 0.774 |  |  | 0.257 |
| No | 27.21% (37.00 / 136.00) | 25.35% (18.00 / 71.00) |  | 33.80% (48.00 / 142.00) | 25.00% (12.00 / 48.00) |  |
| Yes | 72.79% (99.00 / 136.00) | 74.65% (53.00 / 71.00) |  | 66.20% (94.00 / 142.00) | 75.00% (36.00 / 48.00) |  |

**GCS**: Glasgow Coma Scale; **SOFA**: Sequential Organ Failure Assessment; **SAPS II**: Simplified Acute Physiology Score II; **NBPS**: Non-invasive Blood Pressure Systolic; **NBPD**: Non-invasive Blood Pressure Diastolic; **INRPT:** International Normalized Ratio of Prothrombin Time; **PNA**: Pneumonia; **MI**: Myocardial Infarction; **Anti-coa**: Anticoagulation agent; **Anti-pla**: Antiplatelet agent.

**Figure S1**

**
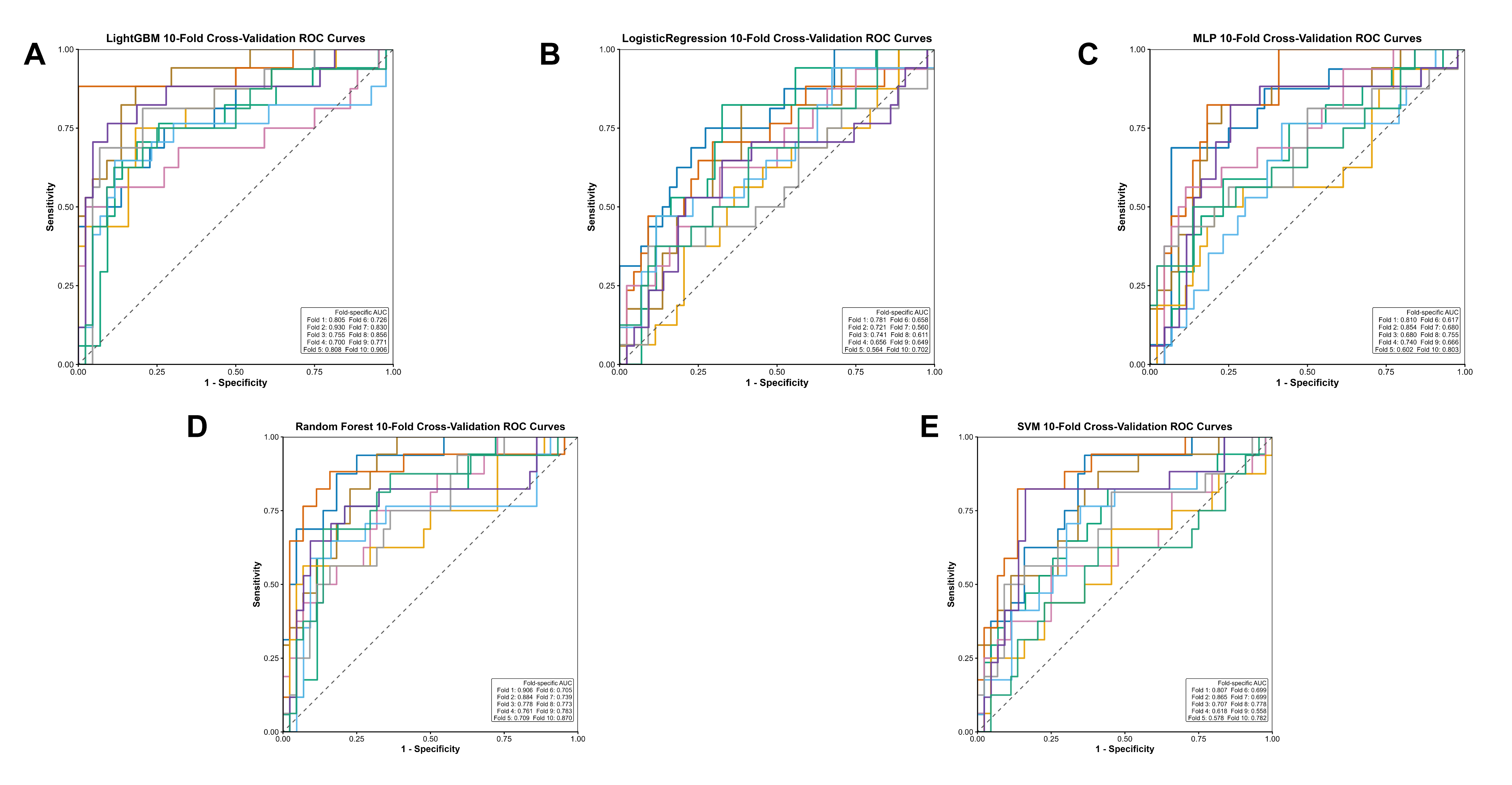
**

**Supplementary Figure S1: 10-fold cross-validation of the five representative models in the internal training set**

**Figure S2**

**
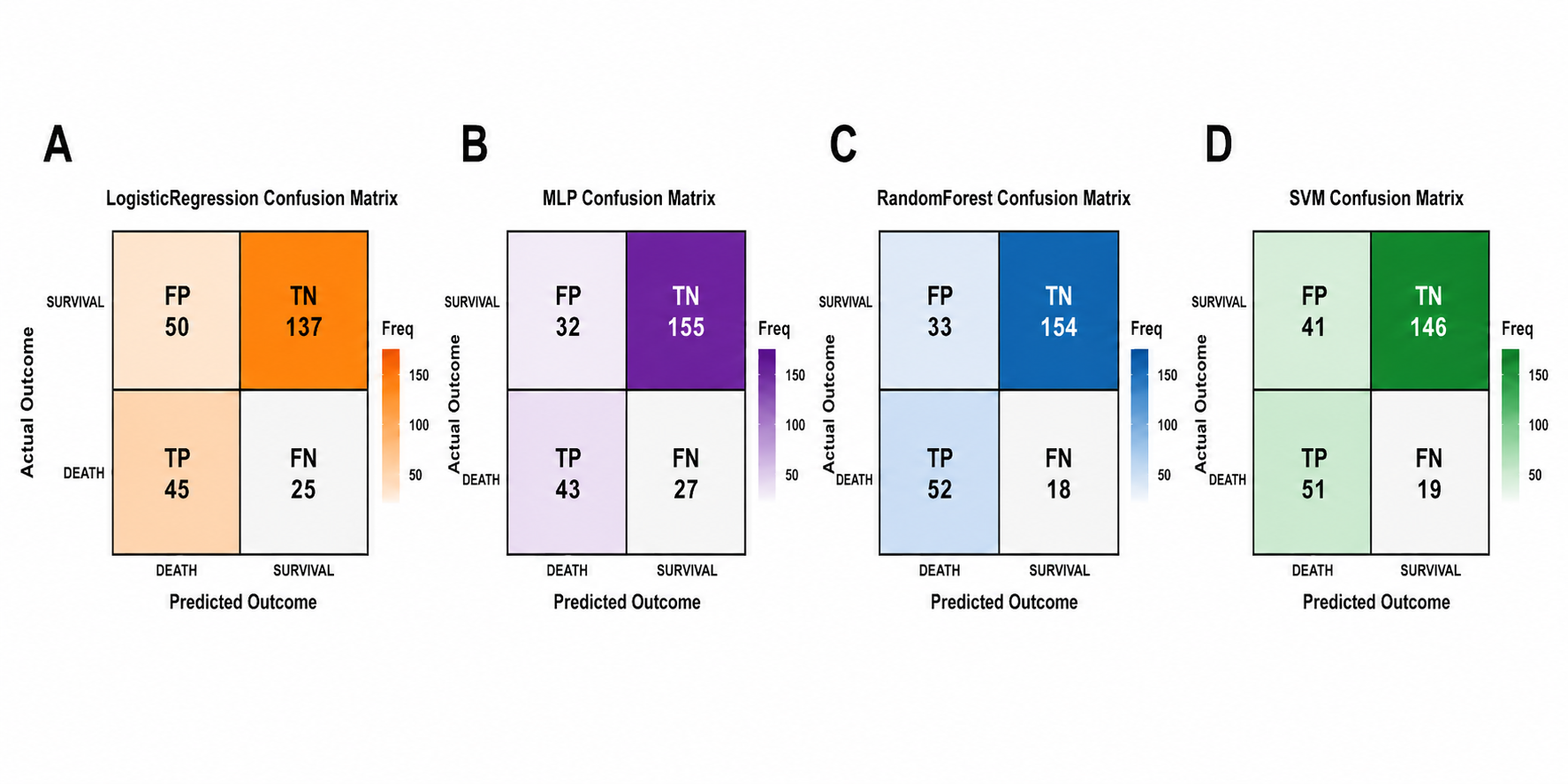
**

**Supplementary Figure S2: The confusion matrix of the model on the internal test set;** (A) Logistic Regression model; (B) Multi-Layer Perceptron; (C) Random Forest; (D) Support Vector Machine

**Figure S3**

**
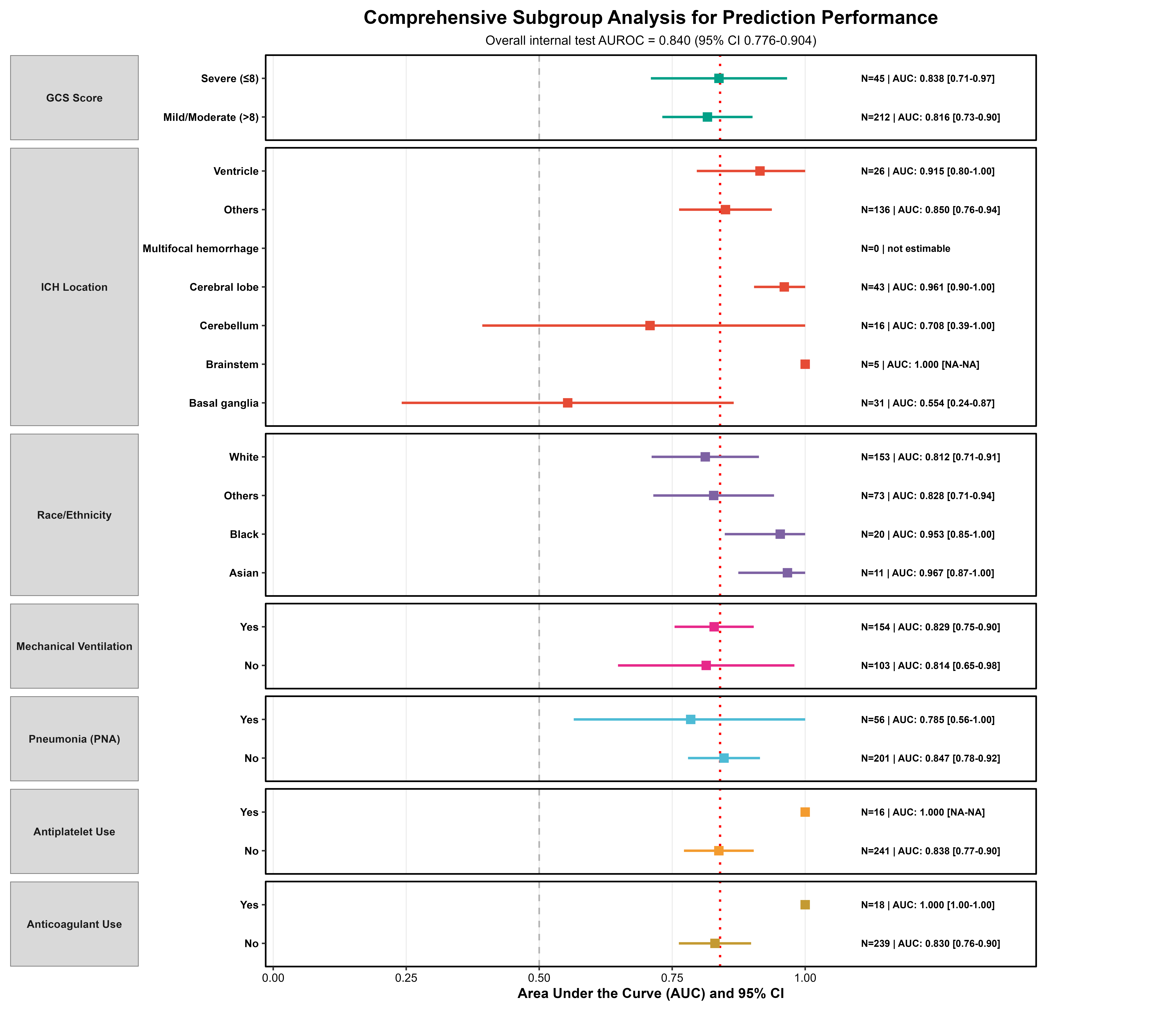
**

**Supplementary Figure S3. Subgroup analysis of the discriminative performance of the optimal model in the internal test set.**

**Figure S4**

**
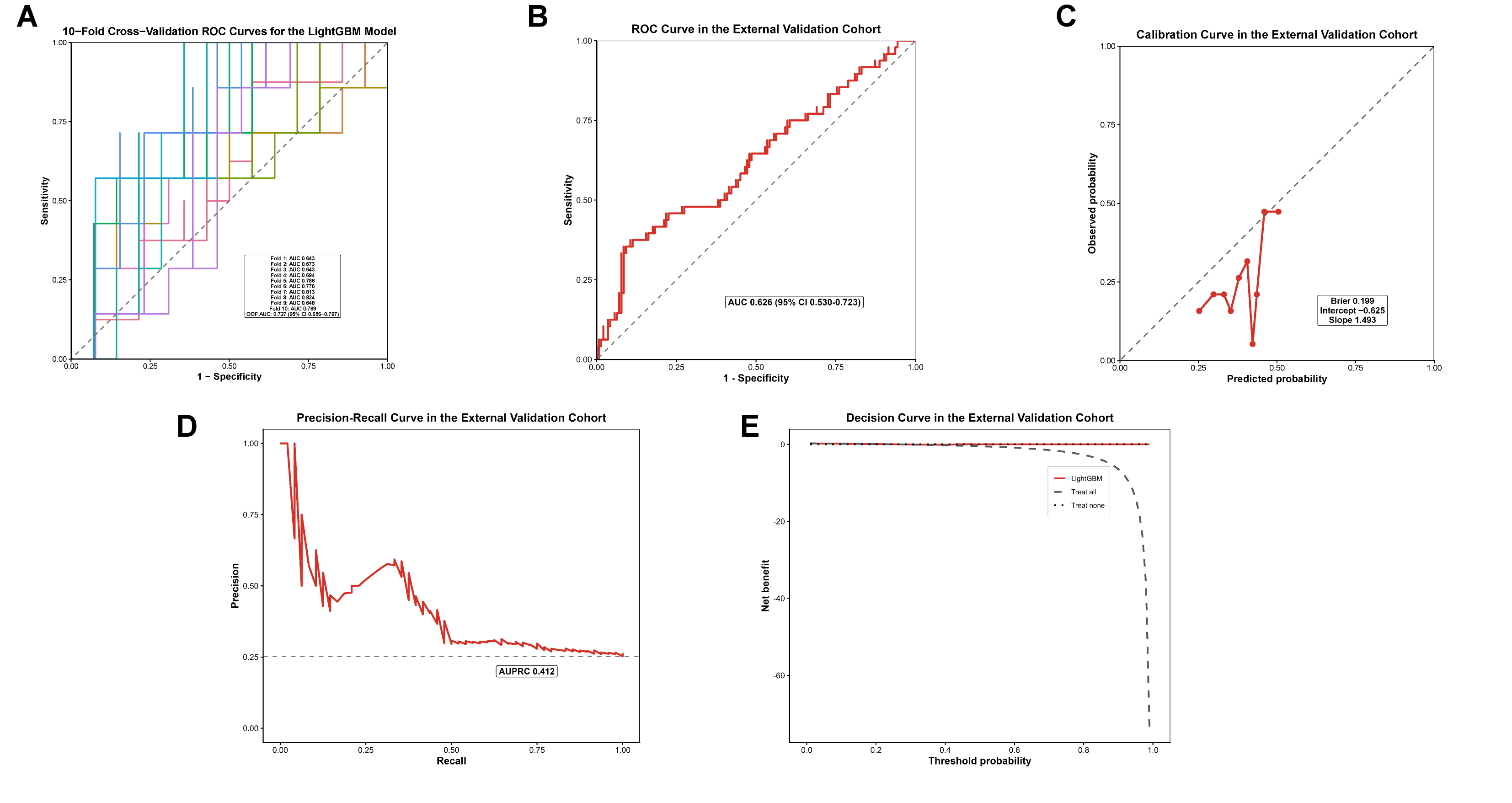
**

**Supplementary Figure S4: Performance of the hematoma-volume-augmented LightGBM model in the hematoma-volume-complete sensitivity analysis.** A sensitivity analysis was performed among patients with available quantitative hematoma volume data. A LightGBM model incorporating the 15 admission-time predictors used in the primary analysis and admission hematoma volume was retrained in the hematoma-volume-complete internal cohort and then evaluated in the hematoma-volume-complete external validation cohort. **(A)** Ten-fold cross-validation receiver operating characteristic curves in the hematoma-volume-complete internal cohort；**(B)** Receiver operating characteristic curve in the hematoma-volume-complete external validation cohort; **(C)** Calibration curve in the hematoma-volume-complete external validation cohort; **(D)** Precision-recall curve in the hematoma-volume-complete external validation cohort; **(E)** Decision curve analysis in the hematoma-volume-complete external validation cohort.

**Figure S5**

**
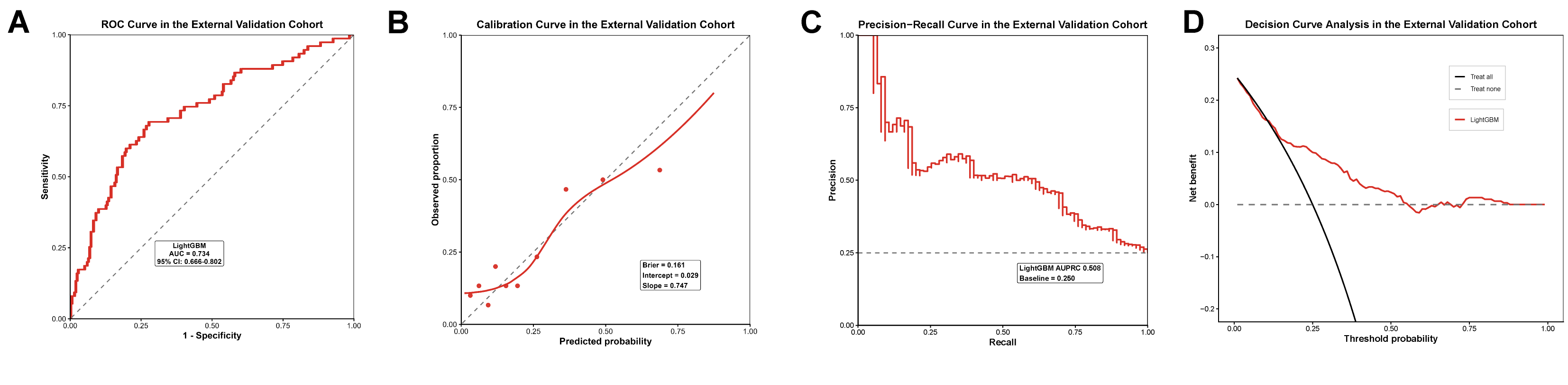
**

**Supplementary Figure S5. External validation of the primary LightGBM model in the hematoma-volume-complete external validation subgroup.** The primary LightGBM model trained using the 15 admission-time predictors was evaluated in the hematoma-volume-complete external validation subgroup. The external validation dataset was used directly without incorporating hematoma volume into model prediction. **(A)** Receiver operating characteristic curve of the LightGBM model, with the AUROC and 95% confidence interval shown. **(B)** Calibration curve based on Platt-calibrated predicted probabilities. The dashed diagonal line represents perfect calibration. **(C)** Precision-recall curve of the LightGBM model. The dashed horizontal line indicates the event prevalence. **(D)** Decision curve analysis showing the net benefit of the LightGBM model across threshold probabilities compared with treat-all and treat-none strategies. LightGBM, light gradient boosting machine; AUROC, area under the receiver operating characteristic curve.

**Figure S6**


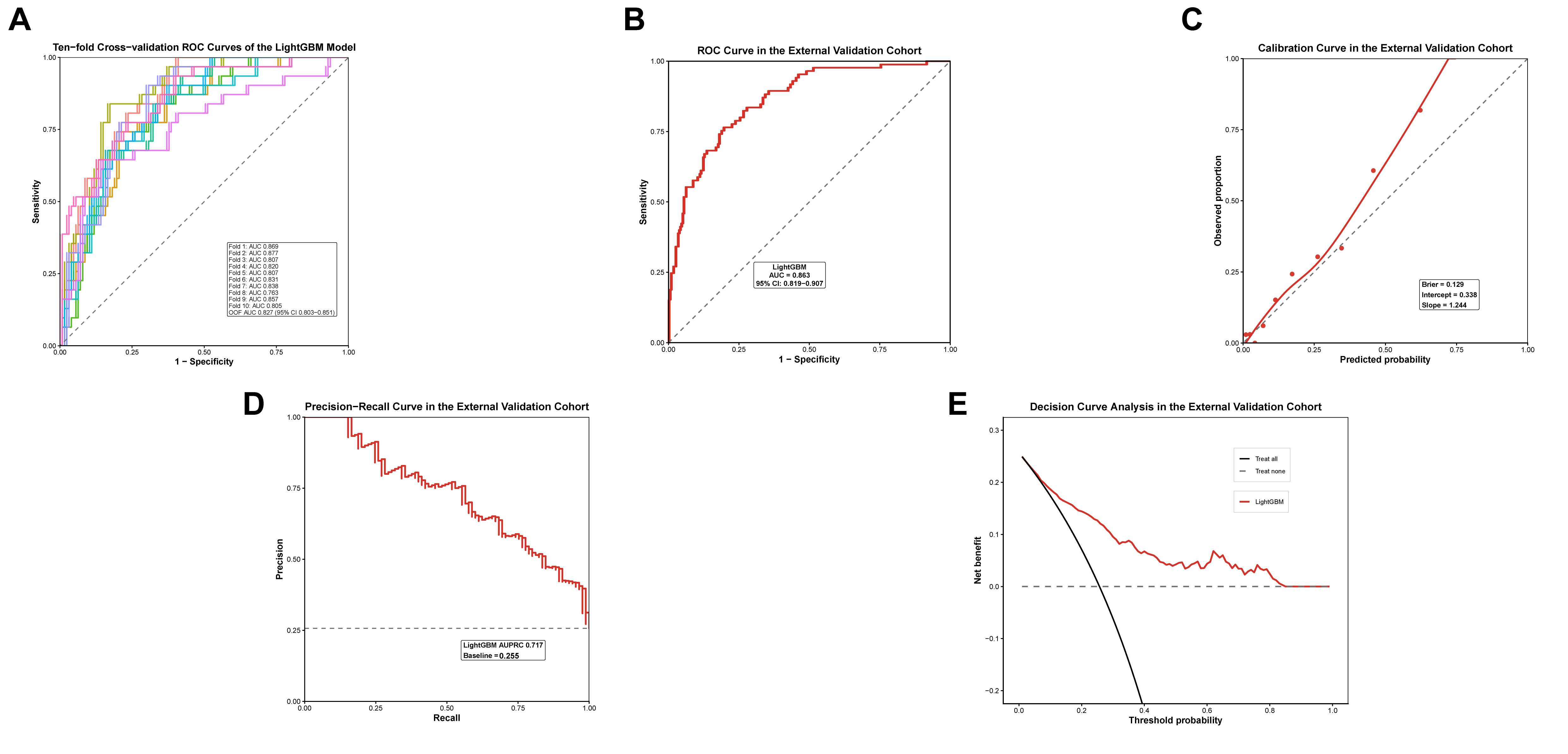


**Supplementary Figure S6. Sensitivity analysis in the expanded thrombocytopenia cohort.**

We included all patients who underwent platelet-count testing within 24 hours after ICU admission, regardless of the number of measurements, and who met the diagnostic criterion for thrombocytopenia (<150×10^9/L), to assess the stability of the primary model and clarify the potential impact of selection bias related to platelet-measurement frequency. The model-development cohort included 1,580 patients, of whom 428 died within 28 days after ICU admission. The external validation cohort included 357 patients, of whom 91 died within 28 days. A LightGBM model using the same 15 admission-time predictors as the primary analysis was retrained in the expanded internal cohort and then evaluated in the external validation cohort. **(A)** Ten-fold cross-validation receiver operating characteristic curves in the expanded internal cohort. **(B)** Receiver operating characteristic curve in the external validation cohort, with the AUROC and 95% confidence interval shown. **(C)** Calibration curve in the external validation cohort after Platt calibration. **(D)** Precision-recall curve in the external validation cohort, with the dashed horizontal line indicating the event prevalence. **(E)** Decision curve analysis in the external validation cohort, comparing the LightGBM model with treat-all and treat-none strategies. LightGBM, light gradient boosting machine; AUROC, area under the receiver operating characteristic curve.

**Figure S7**


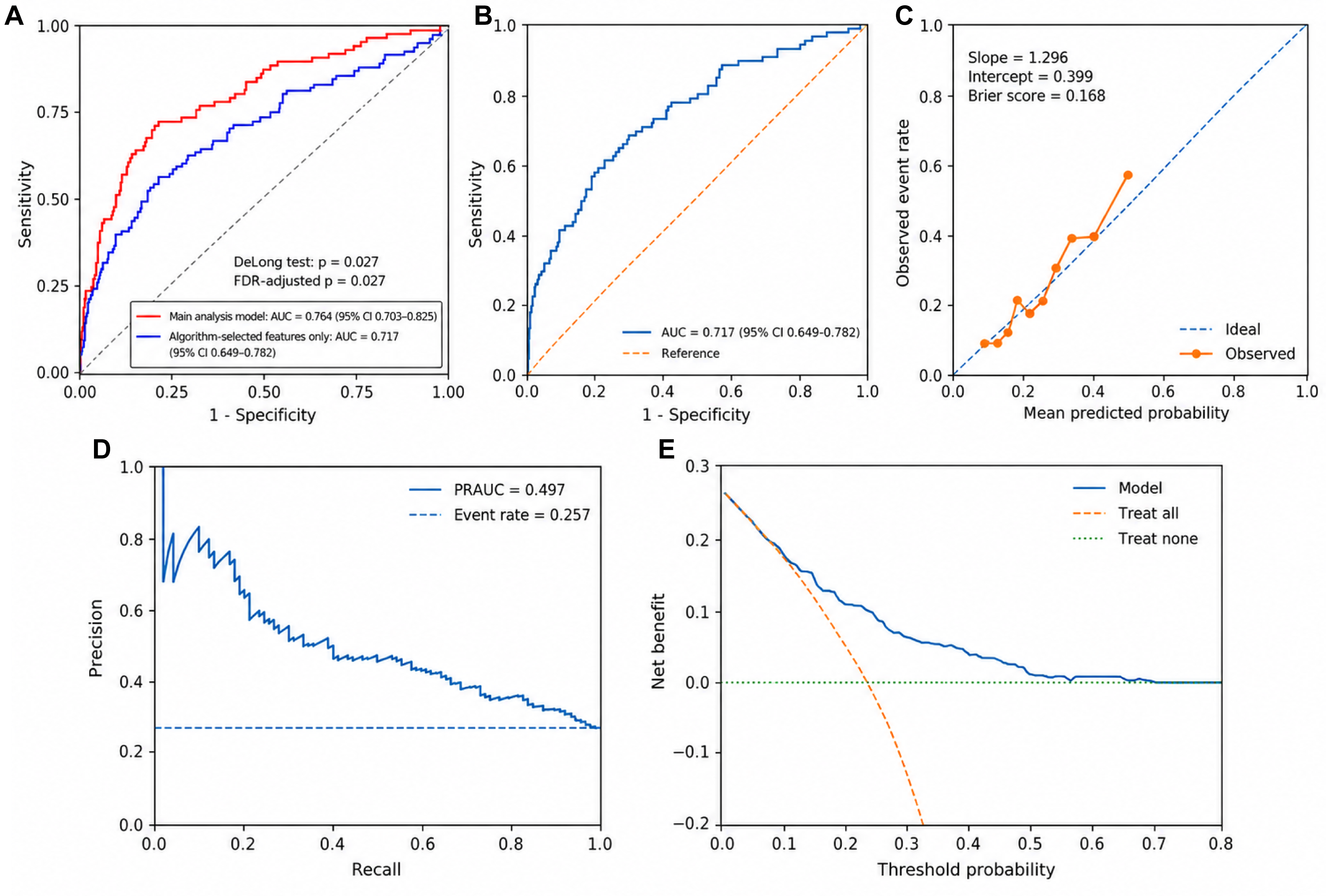


**Figure S7. Sensitivity analysis using algorithm-selected predictors only (the eight predictors jointly selected by both LASSO and Boruta).**

(A) Comparison of receiver operating characteristic curves between the final expert-augmented LightGBM model and the LightGBM model using algorithm-selected predictors only in the external validation cohort. (B) ROC curve of the algorithm-only model. (C) Calibration curve of the algorithm-only model. (D) Precision–recall curve of the algorithm-only model. (E) Decision curve analysis of the algorithm-only model. The final expert-augmented model achieved a higher AUROC than the algorithm-only model (0.764 vs 0.717; DeLong test, P=0.027; FDR-adjusted P=0.027).

**Figure S8**

**
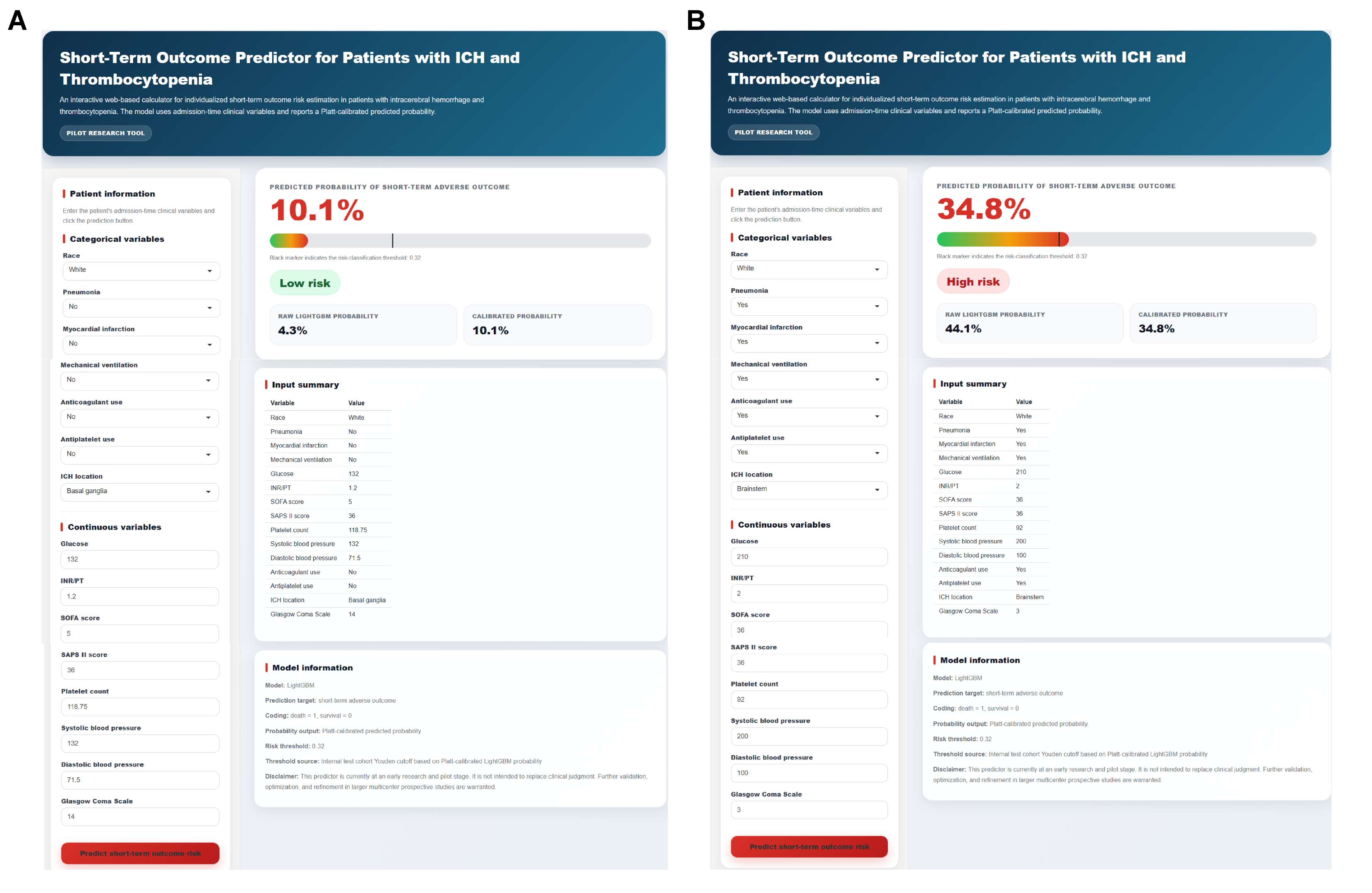
**

**Supplementary Figure S8 Clinical application of our prediction model.** The web application is intended for adult ICU patients with non-traumatic ICH and thrombocytopenia within 24 hours after ICU admission. It provides a Platt-calibrated probability of 28-day all-cause mortality. The reporting threshold was based on the Youden index, but clinical thresholds should be context-specific. **(A)** Interactive interface demonstrating how the model predicts low-risk patients; **(B)** Interactive interface demonstrating how the model predicts high-risk patients.
